# Previous crop and rotation history effects on maize seedling health and associated rhizosphere microbiome

**DOI:** 10.1038/s41598-017-15955-9

**Published:** 2017-11-16

**Authors:** Maria-Soledad Benitez, Shannon L. Osborne, R. Michael Lehman

**Affiliations:** 1 North Central Agricultural Research Laboratory, 2923 Medary Ave., Brookings, SD 57006 USA; 2 Present Address: The Ohio State University, Department of Plant Pathology, 1680 Madison Ave., Wooster, OH 44691 USA

## Abstract

To evaluate crop rotation effects on maize seedling performance and its associated microbiome, maize plants were grown in the greenhouse in soils preceded by either maize, pea, soybean or sunflower. Soils originated from a replicated field experiment evaluating different four-year rotation combinations. In the greenhouse, a stressor was introduced by soil infestation with western corn rootworm (WCR) or *Fusarium graminearum*. Under non-infested conditions, maize seedlings grown in soils preceded by sunflower or pea had greater vigor. Stress with WCR or *F*. *graminearum* resulted in significant root damage. WCR root damage was equivalent for seedlings regardless of soil provenance; whereas *F*. *graminearum* root damage was significantly lower in maize grown in soils preceded by sunflower. Infestation with WCR affected specific microbial taxa (*Acinetobacter*, *Smaragdicoccus*, *Aeromicrobium*, *Actinomucor*). Similarly, *F*. *graminearum* affected fungal endophytes including *Trichoderma* and *Endogone*. In contrast to the biological stressors, rotation sequence had a greater effect on rhizosphere microbiome composition, with larger effects observed for fungi compared to bacteria. In particular, relative abundance of Glomeromycota was significantly higher in soils preceded by sunflower or maize. Defining the microbial players involved in crop rotational effects in maize will promote selection and adoption of favorable crop rotation sequences.

## Introduction

Current intensive agricultural practices include the widespread adoption of two-year maize (*Zea mays* L.) and soybean *(Glycine max* L. Merr.*)* rotations. Even though short rotations provide benefits compared to a continuous cropping system^1^, greater value has been described for more diverse, but little adopted, longer rotation sequences^2,3^. Benefits from diversified crop rotations extend beyond yield, and include increased soil nutrients and organic matter content^4,5^, improved soil structure^6,7^, enhanced soil microbial biomass, increase in microbial diversity and activity^5,8,9^; and disruption of pests, weeds and disease cycles^10–13^. In rotational systems, both the length of the rotation and the plant species included in the rotation contribute to differences in rotational effects on soil characteristics and cash crop benefits^6,11,14^. Some of these rotational effects result from variations in root architecture and rhizodeposition, and influence biogeochemical cycles^15^. Furthermore, both a legacy effect from the field’s past history^16–18^, as well as a direct effect of the crop immediately prior to the cash crop are important^11,19^.

Crop rotation also affects the structure and dynamics of soil and plant-associated microbial communities. Microbial communities are known to respond to host plant identity and genetics, soil characteristics and climatic conditions^20,21^. Microbial species living in close-proximity, or in association with plants are directly influenced by the root’s architecture as well as the chemical characteristics of root exudates^22^. The narrow zone of direct influence of plant roots in soil is known as the rhizosphere. The rhizosphere harbors elevated numbers of active microorganisms compared to the bulk soil, including plant pathogens, plant-beneficial microorganisms and saprotrophs^23,24^. Plant beneficial microorganisms can promote plant health and growth through different mechanisms. These include production of plant-hormone analogs, promotion of systemic resistance to plant pathogens, direct antibiosis against plant pathogens and nutrient acquisition and mobilization^25^. Hence, one of the mechanisms through which diversified crop rotations could benefit crop species is through enrichment of microbial taxa positively affecting crop growth^2^.

A long-term field experiment evaluating different four-year rotation sequences was established in the year 2000 at the Eastern South Dakota Soil and Water Research Farm in Brookings, South Dakota. Rotation sequences in this experiment consider combinations of small grains, legumes and oil-seed crops, as well as cool season and warm season crops. Preliminary data, after 16 years of experiment establishment, showed rotation-sequence-specific benefits at both the soil and plant level. For instance, maize yields tend to be higher in rotations where maize follows pea (*Pisum sativum L*.) and lower in rotations where maize follows soybean. Furthermore, the four-year rotations including sunflower *(Helianthus annus L*.*)* resulted in greater soil carbon accumulation and soil aggregate stability over the 16 year period (Osborne *et al*., unpublished data). Therefore, we hypothesized that maize seedlings growing in soils from rotation sequences which promote greater plant yield and soil health (i.e. those including sunflower and pea) will be less affected by a biotic stressor; and that the effect of the biotic stress is associated with specific changes in maize-associated bacterial and fungal communities. To test this hypothesis, we collected soils from four different rotations, for which either maize, pea, soybean or sunflower preceded maize in the field. In the greenhouse, maize seedlings were grown in these soils and challenged with either western corn rootworm (*Diabrotica virgifera virgifera* LeConte) or *Fusarium graminearum*. Maize plant health and vigor were subsequently measured and rhizosphere bacterial and fungal communities were assessed by amplicon sequencing of ribosomal markers.

## Materials and Methods

### Site description and soil collection

Soils were collected from a long-term research experiment established at the Eastern South Dakota Soil and Water Research Farm in Brookings, South Dakota (44°19′N latitude; 96°46′W longitude). This field experiment evaluates benefits of a variety of no-till, four-year rotation sequences. Rotational treatments were established in the year 2000, in a randomized complete block design with four replications per treatment, and 93 m^2^ plot size. Each crop in a given rotation sequence is present each year. For the purpose of this experiment the following rotation sequences were examined: a) soybean – spring wheat (*Triticum aestivum* L.) – maize - maize, b) soybean – spring wheat – sunflower– maize, c) soybean – spring wheat – pea– maize, and d) oat (*Avena sativa* L.) – winter wheat – soybean – maize. Soils were collected from plots corresponding to the crop preceding maize in each of these rotations (i.e., the third year of the rotation sequence: maize, sunflower, pea and soybean, respectively). Soil collection was conducted in fall 2015, after crop harvest and before ground freezing. Twelve soil cores were sampled at random in each plot, at 0–15 cm depth, with a total of four replicate plots per rotation sequence. Collected field soils were maintained at 4 °C until greenhouse experiment setup.

### Western corn rootworm experiment

Maize plants were grown in 2 L pots containing ~12% of rotation field soil and a potting mixture. In order to minimize disturbance in the experimental field plots, the amount of field soil to be collected and used in the greenhouse experiment was limited. A soil dilution approach was selected in order to test multiple replicates per soil provenance and biotic stressor combinations. The potting mixture was composed of 1:1:1 (m^3^:m^3^) quartz sand (4030 silica sand, 0.45–0.55 mm diameter, Unimin Minnesota Corp, Le Sueur, MN), calcined clay (Turface All Sport Pro, Profile Products, Buffalo Grove, IL), and dry, sieved field soil (Barnes sandy clay loam; fine-loamy, mixed, superactive, frigid Calcic Hapludoll). Prior to use the potting mixture was autoclaved in dry cycle for 80 minutes, twice, and allowed to cool. Soil cores from replicate plots of the same rotation sequences were pooled and thoroughly mixed with a cultivator, immediately prior to mixing with potting mix and experiment setup. All tools were cleaned and disinfested with 70% ethanol in between soils from different rotation treatments. All experiments were performed with untreated seed from maize hybrid 60-01N (Viking, Alberta Lea Seeds, Alberta Lea, MN).

Four maize seeds per pot were sowed prior to western corn rootworm (WCR) infestation. WCR were obtained from a non-diapause colony from the corn rootworm rearing facility of the North Central Agricultural Research Laboratory, USDA, Brookings, SD. WCR inoculum was prepared from one day old eggs suspended in 0.15% agar solution. Four mL of egg suspension, containing ~350 eggs, was dispensed in the middle of each pot. Non-infested pots received 4 mL of plain 0.15% agar solution. Seedling germination was recorded and germinated seedlings were culled in order to keep only one plant per pot throughout the extent of the experiment. Pots were setup in the greenhouse in a randomized design, with 8 pots per rotation field soil (soil provenance) and infestation treatment combination. Pot locations were rotated every three days in the greenhouse. Greenhouse conditions were setup as 16 h days with temperatures at 28 °C day and 18 °C night (with maximum and minimum recorded values at 34 °C and 12 °C for the length of the experiment). Seedlings were evaluated and destructively sampled four weeks after planting (equivalent of vegetative stage 3–4). Root damage due to WCR was scored based on Musick and Suttle’s root damage rating system (1–9 scale), as cited in Oleson *et al*.^26^. In addition, shoot tissue was collected and dried at 60 °C for biomass measurements and plant tissue nutrient analysis. Dried shoot tissue was ground in a Wiley mill, sieved (2 mm), and analyzed for the following nutrients: phosphorous (P), potassium (K), calcium (Ca), magnesium (Mg), zinc (Zn), manganese (Mn), boron (B), iron (Fe) and copper (Cu) by inductively-coupled plasma emission spectrometry (Ag Lab Express, Sioux Falls, SD). Total carbon (C) and nitrogen (N) analysis were performed by dry combustion on a LECO CN 628 analyzer (Leco Corp., St Joseph, MI). Rhizosphere samples, defined as roots plus adhering soils (after shaking), were collected for microbiome analyses at the time of scoring and stored at −80 °C until processing.

### *Fusarium graminearum* experiment

The *F*. *graminearum* isolate used in this experiment was previously recovered from maize fields in South Dakota and was chosen based in its performance on aggressiveness tests (provided by P. Okello and F. Mathew, South Dakota State University)^27^. *F*. *graminearum* inoculum was prepared on double-autoclaved cornmeal-sand substrate^28^. As above, maize seedlings were grown in 2 L pots with or without infestation with *F*. *graminearum*. Each 2 L pot was filled halfway with ~12% field soil - potting mixture (described above), followed by 20 g of *F*. *graminearum* infested cornmeal (or non-infested cornmeal for controls), and filled to the top with the field soil - potting mix. Pots were moistened and five maize seeds were planted at ~2.5 cm depth. Pots were setup in the greenhouse in a randomized design, with 8 pots per soil provenance and infestation treatment combination. Pots were evaluated for germination at day four and time of sampling. All seedlings per pot were scored and destructively sampled two weeks after planting (vegetative stage 2). Root damage was assessed according to an ordinal scale, where 0 corresponds to 0% of seedling tissue visibly damaged and 12 corresponds to 91–100% tissue damaged. The root (and associated soils; i.e. rhizosphere) of one seedling per pot was kept for microbiome analysis and the leftover roots were scanned and processed with WinRhizo software (Regent Instruments Canada Inc) for root architecture measurements. For this, roots were gently rinsed with distilled water to remove excess soil prior to scanning. Measured root variables included: length, diameter, surface area, volume and the numbers of root tips, forks and crossings; as well as root length at specific diameter size classes. Diameter size class length contribution data was converted to percent, where each individual diameter size class contributes to a percent of the total root length. Maize shoot tissue was also collected per pot and processed for biomass and nutrient content estimation, as described above. Greenhouse conditions were the same as for the WCR experiment.

### Library preparation for amplicon sequencing

At the time of sampling, the rhizosphere from one maize plant per pot was chopped into <0.5 cm pieces, mixed and stored at −80 °C until processing. Prior to DNA extraction, approximately 0.2 g wet weight of rhizosphere sample was freeze-dried for 4–6 hours in a FreeZone Freeze Dry System (Labconco, Kansas City, MO) and ground with two 5/32′′ stainless-steel beads in a Mini Bead Beater (Bio Spec Products, Bartlesville, OK) for 30 s. Freeze-ground tissue was then processed with Qiagen’s DNeasy® Plant Mini kit (Qiagen, Germantown, MD) following manufacturer’s recommendations. Library preparation was performed using a two-step PCR protocol, based on Illumina’s Tech. Note 15044223^29^. The 16S V4 and ITS2 regions were used for bacterial and fungal amplicon sequencing of maize rhizosphere samples, respectively. For this, primers “515f modified” and “806r modified”, from Walters *et al*.^30^, and ITS3mix1 to 5 and ITS3mix10, as a forward mix, with ITS4ngs as reverse^31^, each containing an overhang tag for Nextera kit indexing, were used for amplification of the 16S and ITS2 regions, respectively (Supplementary Table 1). Different DNA controls were included in amplification, indexing and sequencing reactions. Positive controls were comprised of total DNA from 12 different isolates from a range of taxonomic groups, for each bacteria and fungi. For bacteria, these included isolate DNA from Proteobacteria (Alpha and Gamma), Firmicutes, Actinobacteria, Bacteroidetes and Acidobacteria. For fungi it included a mixture of Ascomycota (including the *F*. *graminearum* isolate used in the greenhouse experiments), Basidiomycota, Zygomycota and Glomeromycota isolates. DNA extraction controls and non-template samples were included as negative controls. Gene-specific amplification was performed using KAPA HiFi 2x PCR Ready Mix (Kapa Biosystems, Wilmington, MA) in 12.5 µL reactions containing 0.2 µM each primer and 1 µL DNA template; and an amplification cycle of 94 °C initial denaturation, followed by 25 cycles of 30 s at 98 °C, 30 s at 55 °C and 1 min at 72 °C, with a final 5 min extension at 72 °C. Indexing and Illumina adapters were added in a second amplification reaction using the dual indexing system of Illumina Nextera XT Indexing kit v2 (Illumina, San Diego, CA). Specifically, in a 25 µL reaction containing KAPA HiFi 2x PCR Ready Mix, 2.5 µL each index and 2.5 µL of PCR product. The indexing amplification cycle consisted of 94 °C initial denaturation, followed by 10 cycles of 30 s at 98 °C, 30 s at 55 °C and 1 min at 72 °C, with a final 5 min extension at 72 °C. Indexed PCR products were cleaned with Agencourt AMPure XP beads (Beckman Coulter, Brea, CA) at a 1:1 and 0.8:1 bead:template ratio for 16S and ITS2 amplicons, respectively. Cleaned amplicons were quantified using Quant-iT Picogreen ds DNA Assay kit (Life Technologies/Thermo Fisher Scientific, Walthman, MA) and amplicons were pooled in equimolar concentrations. The pooled sample was cleaned with Agencourt AMPure XP beads at a 0.8:1 bead:template ratio and submitted for a 2 × 300 Illumina MiSeq sequencing run at the University of Minnesota Genomics Center, Microbiome Sequencing Services (University of Minnesota, St. Paul, MN).

### Sequence data processing

Pair-ended de-multiplexed fastq reads were merged and processed following UPARSE pipeline in usearch v8.1^32^. Specifically, after merging of paired-end reads, gene specific primers were removed using cutadapt v1.1^33^, followed by quality filtering, de-replication, reference based chimera checking, clustering into Operational Taxonomic Units (OTUs) and mapping into a OTU-to-sample table (Supplementary Methods). For bacteria, taxonomic assignment was performed with the *utax* algorithm, based on the ribosomal database project training set 15^34^. Classification and taxonomy assignments for groups of interest were revised based on the List of prokaryotic names with standing nomenclature^35^. For fungi, taxonomic assignment was performed in Qiime v1.9.1^36^ using UNITE ITS^37^ release v7.1 as a reference database. Additional non-target sequences and chimeras were identified and removed and alpha diversity metrics were calculated from sequence read counts using R’s phyloseq package^38^. Sequence count normalization was performed in Qiime, using metagenomeSeq’s CSS (cumulative sum scaling^39^), as incorporated in the *normalize_data*.*py* script, per-experiment, after filtering-out OTUs present in less than 5 samples and/or with sequence counts lower than 10. OTU-by-sample tables were merged with plant vigor and shoot nutrient data for further analysis in R^40^.

Prediction of functional bacterial and fungal diversity within 16S and ITS2 sequence libraries were performed using PICRUSt^41^ and FUNGuild^42^, respectively. PICRUSt predicts the potential metagenomic gene content of a 16S amplicon library, based on genomic information of bacteria represented within the greengenes 16S database^41^. For PICRUSt, the taxa by sample matrix must be generated using Qiime’s closed reference OTU picking with greengenes database v 13.5^41,43,44^ and sequence counts normalized to 16S copy number prior to analysis. PICRUSt was ran as part of the bioBakery tools repository^45^. FUNGuild, on the other hand, assigns trophic mode and guild to fungal taxa, based on comparison to a curated database of fungal life styles and use of resources. Trophic mode refers to the mechanisms through which organisms obtain resources, hence potentially providing information on the ecology of such organisms^42^. Functional guild assignments through FUNGuild are based on taxonomy, and are possible only if taxa has been classified at the genus level^42^ or if taxa belong to a fungal group with exclusive lifestyle (e.g. Glomeromycota). Input data for FUNGuild was the CSS normalized taxa-by-sample matrix, after filtering for low incidence and abundance counts (see above).

### Statistical analyses

The effect of preceding crop and infestation with WCR or *F*. *graminearum* on maize germination, shoot biomass and nutrient content was evaluated using the Kruskal-Wallis test as incorporated in R’s agricolae package v 1.2-2^46^. The relationships between microbial community composition, preceding crop and infestation effects were visualized using correspondence analysis (CA), and further correlations with seedling vigor measurements (i.e. seedling biomass, root measures and plant nutrient content) were evaluated through canonical correspondence analysis (CCA). Correspondence analysis and CCA were performed in R’s vegan package v2.4-1^47^ from normalized sequence abundance data. Additional visualization of site and taxa ordination in CA, was performed in R’s phyloseq package. Tests of differential abundance of taxa in response to previous crop and infestation treatments were also performed in phyloseq, using the F-test incorporated in the *mt* command which corrects for multiple hypothesis testing by control for false discovery rate^38^. Further, multiple comparison tests of taxa differentially abundant across treatments were performed using the Kruskal-Wallis test. Differential abundance and multiple comparison tests were performed for OTUs and for taxa aggregated at different taxonomic ranks. Significance is reported as p < 0.1.

### Data Availability

Data analyzed during this study are included in this article and accompanying Supplementary Information. Raw sequences generated in this work have been deposited in NCBI’s Sequence Read Archive under BioProject number PRJNA385957.

### Disclaimer

Mention of trade names or commercial products in this publication is solely for the purpose of providing specific information and does not imply recommendation or endorsement by the U.S. Department of Agriculture. USDA is an equal opportunity provider and employer.

## Results

### Previous crop and infestation effects on maize seedlings

Soil infestation with WCR resulted in major root damage, regardless of soil provenance (Table 1). Maize plants grown under infested conditions had up to 7% less shoot biomass than their non-infested counterparts, in particular for the pea rotation treatment. In addition, maize plants grown in soils preceded by sunflower or pea had 14–19% greater shoot biomass than those preceded by maize or soybean, regardless of infestation. The effects of WCR on maize plants were also detected in plant nutrient content (Supplementary Table 2). Maize plants grown under infested soils had lower nutrient content for all the elements studied, except Zn, Cu, and Fe; whereas C content was higher in plants under WCR infestation. Soil provenance had an effect on plant P, Ca, Mn, B and Cu content, with maize plants grown in soils preceded by soybean exhibiting the lowest plant nutrient concentrations. Maize plants grown in soils preceded by sunflower consistently had the lowest (or second lowest) percent difference in nutrient content in response to infestation (for plant P, K, Ca, Mg, Mn and B, in particular).

**Table 1:** Soil provenance and infestation effects on maize seedlings grown in soils originating from four four-year rotation treatments, each with a different crop preceding maize.

Soil provenance	Western corn rootworm								*Fusarium graminearum*									
	Total germination^a^		Shoot dry weight (g)^b^			Root damage score^c^			Germination day 4^d^			Total germination^a^		Shoot dry weight (g)^e^		Root damage score^f^		
**Non-infested**																		
Maize	5.00	±0.0	2.02	±0.20	bc	0.38	±1.06	B	4.88	±0.35	aA	5.00	±0.0	0.24	±0.02	1.16	±0.27	bB
Pea	5.00	±0.0	2.35	±0.27	aA	1.13	±1.55	B	3.50	±1.20	bB	5.00	±0.0	0.23	±0.01	1.34	±0.19	aB
Soybean	4.88	±0.35	1.90	±0.19	c	0.38	±1.06	B	4.25	±0.71	b	5.00	±0.0	0.23	±0.02	1.19	±0.26	bB
Sunflower	5.00	±0.0	2.22	±0.21	ab	0.75	±1.39	B	4.88	±0.35	a	5.00	±0.0	0.24	±0.02	1.63	±0.42	aB
**Infested**																		
Maize	4.75	±0.71	1.96	±0.40	b	6.63	±0.52	A	3.88	±1.55	B	4.88	±0.35	0.24	±0.03	2.39	±0.44	aA
Pea	5.00	±0.0	2.18	±0.20	aB	6.13	±0.83	A	4.50	±0.53	A	4.88	±0.35	0.24	±0.02	2.19	±0.26	aA
Soybean	5.00	±0.0	1.80	±0.06	b	6.13	±0.64	A	3.63	±1.19		4.88	±0.35	0.23	±0.02	2.41	±0.27	aA
Sunflower	5.00	±0.0	2.10	±0.12	a	6.50	±0.76	A	4.75	±0.46		4.75	±0.46	0.24	±0.01	2.08	±0.41	bA
*Soil effect*				***						**								
*Infestation*				*			***						**				***	

^a^Total number of germinated seedlings per pot. Five seeds were planted per pot, per soil origin and infestation treatment.

^b^Value represents mean of n = 8 pots per soil origin and infestation treatment combination and +/− standard deviation. Means followed by different letters are significantly different at *p* < 0.1 after Kruskal-Wallis test. Absence of letter means no significance was detected across comparisons. Comparisons between rotation sequences, within infestation level are shown by lower case letters. Comparisons between infested and non-infested counterparts of the same rotation treatment are shown in upper case letters. Significant effects of soil provenance or infestation are shown as *p < 0.1, **p < 0.05, ***p < 0.001.

^c^Musick and Suttle’s root damage rating (1–9 scale) for WCR, as cited in Oleson (*et al*. 2005).

^d^Number of germinated plants per pot four days after planting.

^e^For *Fusarium* experiment, plant measures were averaged per seedling per pot, with up to 5 seedlings germinated and scored in one pot.

^f^Root damage score according to ordinal scale, where 0 is 0% of seedling tissue damaged and 12 91–100% tissue damaged.

Soil infestation with *F*. *graminearum* resulted in approximately 5% difference in plant germination (Table 1). Root damage was greater in maize plants grown in soils infested with *F*. *graminearum*, however root damage was generally low, representing less than 10% damage of total root. An *F*. *graminearum* infestation effect on plant nutrient content (Supplementary Table 2) was observed for plant P, S, Zn and N, with nutrient content being up to 20% greater in infested samples, in particular when considering pea as preceding crop. As no fertilizer was applied in these experiments, maize seedlings exhibited a trend towards deficiency of Ca, Mg, Zn, Cu and N.

The effects of soil provenance and infestation with *F*. *graminearum* on maize roots were also evaluated through measurements of root morphology and architecture. Under non-infested conditions, soil provenance affected root length and diameter of maize seedlings (Table 2), with roots from seedlings grown in soils preceded by pea being 7–17% shorter than when grown in soils preceded by sunflower and maize. Root diameter, however, was greater (11%) when maize was grown after pea than after sunflower or maize. Infestation effects were observed in root surface area, root volume and the number of forks, with a significant increase for all three measures. When considering root length at different diameter classes, 70–77% of the root length fell within the smallest diameter class (0–0.5 mm) for all treatments, however diameter class distribution differed across soil provenance. Maize seedlings grown in non-infested soils preceded by sunflower had four percent more of its root length represented by the smallest diameter class (0–0.5 mm), compared to the seedlings grown in non-infested soils preceded by pea (Supplementary Figure 1). Conversely, maize seedlings grown in non-infested soils preceded by pea had greatest percent of its root length represented by bigger diameter size classes (2.5–3 mm, 3–3.5 mm and 4–4.5 mm). Infestation effects on percent of root length represented by each diameter size classes was observed in soils preceded by maize or pea only. If preceded by maize, infestation with *F*. *graminearum* resulted in significantly lower percent root length at the smallest size class (and higher percent at the bigger classes). For soils preceded by pea, however, infestation resulted in significantly higher percent root length at the smallest size class (and lowest percent at the bigger classes).

**Table 2:** Root architecture measurements^a,b^ of maize seedlings grown in soils from four different four-year rotation treatments, each with a different crop preceding maize, with and without infestation with *Fusarium graminearum*.

Soil provenance	Root length (cm)			Root surface area (cm^2^)		Root diameter (mm)			Root volume (cm^3^)			No. root tips		No. crossings		No. forks	
**Non-infested**																	
Maize	744.1	±68.3	a	113.3	±8.1	0.24	±0.02	b	1.38	±0.13		1674	±250	1302	±204	6334	±737
Pea	653.1	±47.2	bB	109.5	±6.3	0.27	±0.01	a	1.47	±0.11		1674	±183	11689	±159	6040	±543
Soybean	716.9	±69.2	ab	112.3	±8.6	0.25	±0.03	ab	1.43	±0.24		1948	±447	1262	±202	6272	±445
Sunflower	766.1	±138.1	a	113.5	±17.7	0.24	±0.02	b	1.35	±0.24	B	1939	±219	1441	±437	6706	±1644
**Infested**																	
Maize	739.2	±106.7		120.6	±18.7	0.27	±0.05		1.58	±0.33		1804	±157	1323	±246	6814	±1042
Pea	740	±76.0	A	115.7	±9.0	0.26	±0.03		1.45	±0.13		1841	±386	1335	±220	6703	±734
Soybean	743.9	±154.9		122.8	±17.3	0.28	±0.03		1.64	±0.24		2087	±689	1323	±299	6935	±1104
Sunflower	802.1	±96.3		122.7	±9.4	0.24	±0.05		1.51	±0.14	A	1850	±184	1521	±267	7159	±853
*Soil effect*						**										**	
*Infestation*				**					**							**	

^a^Value represents mean of n = 8 pots (+/− standard deviation) per soil origin and infestation treatment combination. Root measures were averaged per seedling per pot, with up to 4 roots measured by pot. Means followed by different letter are significantly different at *p* < 0.1 after Kruskal-Wallis test. Absence of letter means no significance was detected across comparisons. Comparisons between rotation treatments, within infestation level are shown by lower case letters. Comparisons between infested and non-infested counterparts of the same rotation treatment are shown in upper case letters. Significant effects of soil provenance or infestation are shown as *p < 0.1, **p < 0.05, ***p < 0.001.

^b^As determined through root scanning and processing with WinRhizo software.

### Fungal and bacterial community responses to soil provenance and infestation

Individual DNA extracts from maize rhizosphere samples for both experiments were used for amplicon sequencing of the bacterial (16S) and fungal (ITS2) rhizosphere-associated microbial communities. The number of raw sequences recovered through an Illumina MiSeq 2 × 300 run were 19,148,934 and 13,282,922 for 16S and ITS2 amplicons, respectively, from which 40–70% of sequences paired. Pair-end reads of greater than 200 bp were used for delimiting OTU with all data aggregated for both experiments. Normalization and statistical analysis were performed at the experiment level and summaries of sequencing results and OTU calling pipeline are presented in Supplementary Table 3. In addition, summaries of individual sample data at the family level are shown in Supplementary Figures 2 to 5. Bacterial and fungal OTU richness, as measured by Chao1 index, recovered from maize rhizosphere was soil provenance dependent, with consistent trends across experiments toward lower diversity in maize rhizosphere grown in soils preceded by pea or soybean, compared to plants grown in soils preceded by maize or sunflower (Table 3). Infestation with *F*. *graminearum* significantly increased bacterial sequence diversity in maize plants preceded by pea.

**Table 3:** Estimates of bacterial and fungal operational taxonomic unit (OTU)^a^ richness^b^ recovered from maize rhizosphere of seedlings grown in soils from four different four-year rotation treatments under infestation with western corn rootworm or *Fusarium graminearum*.

Soil provenance	Western corn rootworm					*Fusarium graminearum*					
	Bacteria Chao1			Fungi Chao1		Bacteria Chao1			Fungi Chao1		
**Non-infested**											
Maize	2849.7	±299.6^c^		154.6	±45.0	3569.5	±287.7	a	304.3	±61.4	a
Pea	2653.6	±247.5		126.5	±36.2	2708.7	±426.3	cB	224.6	±60.7	b
Soybean	2862	±264.8		135.7	±27.2	3138.8	±467.9	b	289	±44.5	a
Sunflower	2859.7	±352.0		133	±35.1	3361.97	±431.5	ab	285.1	±51.9	a
**Infested**											
Maize	2873.5	±299.1	ab	142.2	±45.0	3567.2	±510.2		288.4	±110.2	
Pea	2567	±144.3	c	140.5	±42.4	3424.4	±536.4	A	257.8	±45.4	
Soybean	2669.3	±271.7	bc	146.6	±31.7	3166.4	±516.8		281.9	±90.3	
Sunflower	3039.1	±279.0	a	140.3	±52.2	3218.7	±556.1		282.6	25.5	
*Soil effect*		**					**		*		
*Infestation*											

^a^Bacterial and fungal OTU were recovered through amplicon sequencing of 16S and ITS2 ribosomal regions, respectively. OTU calling was performed using USEARCH/UPARSE algorithms.

^b^Chao1 richness estimate was calculated from sequence counts after OTU calling and filtering of chimeric and non-target OTUs.

^c^Value represents mean of n = 8 rhizosphere samples per soil origin and infestation treatment combination (+/− standard deviation). Means followed by different letter are significantly different at *p* < 0.1 after Kruskal-Wallis test. Absence of letter means no significance was detected across comparisons. Comparisons between rotation treatments, within infestation level are shown by lower case letters. Comparisons between infested and non-infested counterparts of the same rotation treatment are shown in upper case letters. Significant effects of soil provenance or infestation are shown as *p < 0.1, **p < 0.05, ***p < 0.001.

The OTU corresponding to *F*. *graminearum* used as inoculum in these experiments was recovered from both infested and non-infested maize plants, being up to six times more abundant in infested plants (Supplementary Table 4). Identity of this OTU was confirmed based on sequencing of a positive control sample, which included a known mixture of individual fungal taxa. Further analyses of soil origin and infestation effects on fungal communities associated with maize seedlings in this experiment were performed after removal of the *F*. *graminearum* OTU.

**Figure 1:**
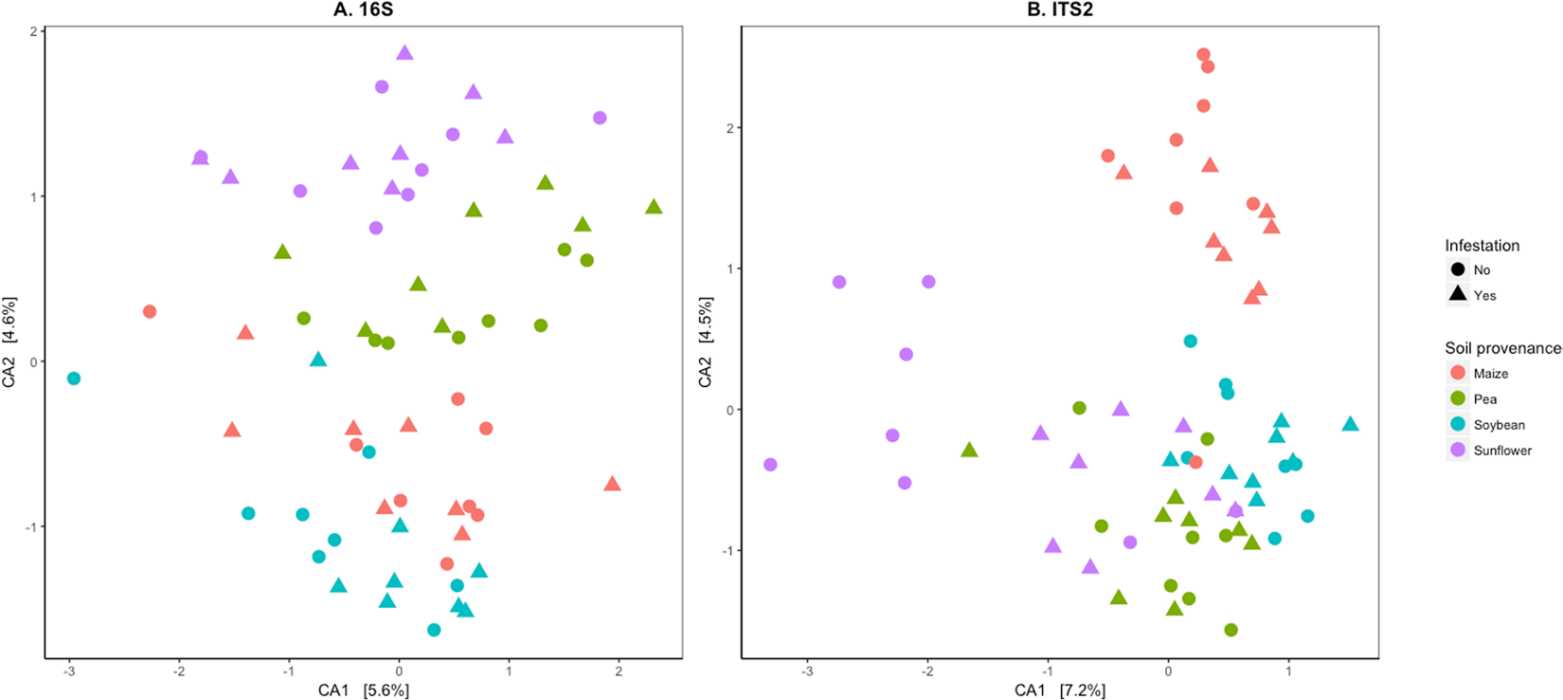
Maize rhizosphere-associated bacterial and fungal community responses to soil provenance and infestation with western corn rootworm. Ordination plots of analyzed samples were generated based on correspondence analysis (CA) of (**A**) bacterial 16S and (**B**) fungal ITS2 OTU by sample matrices. Bacterial and fungal communities were assessed through amplicon sequencing of the 16S and ITS2 regions of the ribosomal DNA, respectively. N = 8 rhizosphere samples per soil provenance (colors) and infestation (shape) treatment combination. The percentage of variation explained by each axis is shown.

**Figure 2:**
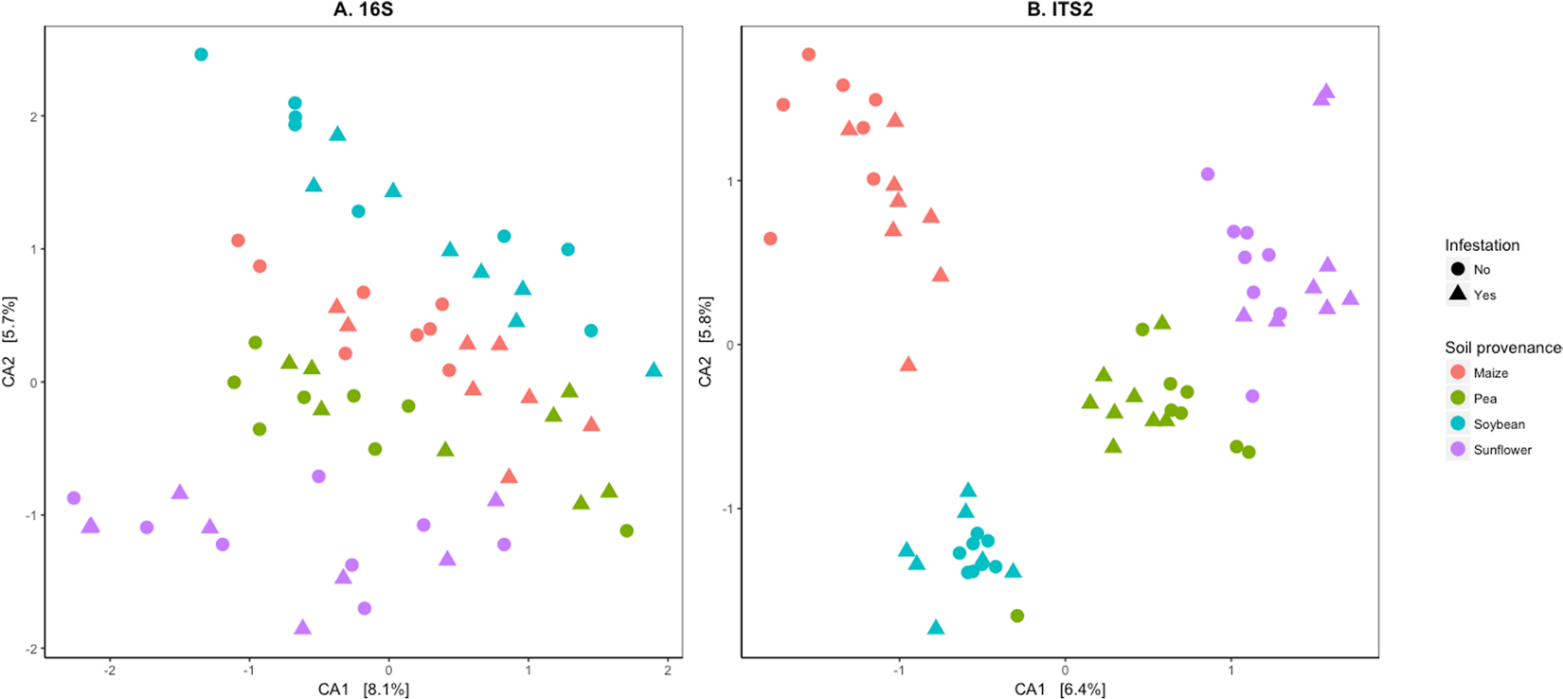
Maize rhizosphere-associated bacterial and fungal community responses to soil provenance and infestation with *F*. *graminearum*. Ordination plots of analyzed samples were generated based on correspondence analysis (CA) of (**A**) bacterial 16S and (**B**) fungal ITS2 OTU by sample matrices. Bacterial and fungal communities were assessed through amplicon sequencing of the 16S and ITS2 regions of the ribosomal DNA, respectively. N = 8 rhizosphere samples per soil provenance (colors) and infestation (shape) treatment combination. The percentage of variation explained by each axis is shown.

The relative effects of soil provenance and infestation on maize rhizosphere-associated bacterial and fungal communities, as evaluated by 16S and ITS2 sequences, were assessed through correspondence analysis and ordination (Figs 1 and 2). In general, bacterial and fungal communities from the same soil provenance clustered closer together in the ordination plots, with comparatively minor influences of WCR (Fig. 1) or *F*. *graminearum* infestation (Fig. 2) on community similarities. Both bacterial and fungal maize rhizosphere communities in seedlings grown in soils preceded by sunflower tended to cluster apart from those grown after maize.

The most abundant bacterial taxa, at the phylum level, were the Proteobacteria, followed by Acidobacteria, Actinobacteria, Bacteroidetes, Verrucomicrobia and Planctomycetes. The Proteobacteria were twice as abundant as the second most-abundant phyla in all samples (Supplementary Table 5). Maize rhizosphere samples grown in soils preceded by pea had a tendency to exhibit the lowest bacterial abundance. A small number of individual bacterial taxa, at the genus level (~10% of the genera recovered) or higher taxonomic rank, showed a consistent effect of soil provenance across experiments (Supplementary Table 6). The Alphaproteobacteria genera *Sphingobium*, *Dongia*, *Rhizobium*, and *Roseomonas*, were most abundant in samples preceded by sunflower (followed by those preceded by maize). Similarly, *Sphingomonas* (Alphaproteobacteria) and members of the phylum Chloroflexi were more abundant in samples preceded by maize or sunflower, compared to those preceded by soybean or pea. Taxa within the candidate division WPS-1, Acidobacteria class GP1 and the Gammaproteobacteria *Rhodanobacter* appeared to be associated more with samples preceded by either maize or soybean, whereas Deinococcus-Thermus taxa were more abundant in samples preceded by soybean (Supplementary Tables 5 and 6). If considering WCR infestation effects only, no individual bacterial OTU were differentially abundant across treatments. At the genus level, however, *Smaragdicoccus* (Actinobacteria, Actinomycetales, Nocardiaceae) and *Acinetobacter* (Gammaproteobacteria, Pseudomonadales, Moraxellaceae) were more abundant in infested maize rhizosphere, whereas *Aeromicrobium* (Actinobacteria, Actinomycetales, Nocardiaceae) was more abundant in non-infested maize rhizosphere (Supplementary Table 7). *Acinetobacter* was 40 times more abundant in infested rhizospheres compared to non-infested, representing the greatest change observed in response to WCR infestation in this study. Similarly, no significant differences were observed in relative abundance of individual bacterial OTU in response to infestation with *F*. *graminearum*. The genera *Clostridium* (Firmicutes, Bacilli, Bacillales, Clostridiaceae) and *Roseimicrobium* (Verrocomicrobia), had greater sequence abundance in non-infested soils compared to infested soils, whereas the class Anaerolinea (Chloroflexi) was significantly more abundant in infested soils (Supplementary Table 8).

For fungi, the most abundant phyla recovered was the Ascomycota, followed by the Zygomycota (Supplementary Figure 6). As for the bacteria, the lowest abundance of fungal taxa was observed in plants preceded by pea (Supplementary Table 9). Significant differences in relative abundance in response to soil provenance were observed for individual fungal OTUs, representing ~10% of the recovered fungal OTUs. For instance, some of the taxa differentially more abundant in maize rhizosphere preceded by sunflower include members of the order Capnodiales, as well as individual OTUs within the Pleosporales (OTU116) and Mortierellales (OTU109). Whereas for those preceded by soybean, members of the Mucorales (Zygomycota), such as *Gongronella* sp. (OTU255) and *Cunninghamella* sp. (OTU86) were most abundant, as well as some chytrids, including *Rhizophydium* spp. (OTU371 and OTU689) and unknown Chytridiomycota species (OTU217). Within the Ascomycota, OTUs belonging to Helotiales (OTU118), Pleosporales (OTU286, genus *Periconia*) and Hypocreales (OTU75, family Nectriaceae) were also predominant in samples preceded by soybean. Fungal taxa most abundant in samples preceded by maize included two Pleosporales OTUs, a *Drechslera* OTU (OTU15) and an unknown Pleosporales (OTU45; Supplementary Table 8). At the phylum level, Glomeromycota were up to three times more abundant in maize rhizosphere when grown in soils preceded by sunflower or maize, with the genus *Rhizophagus* following a similar pattern. Contrary to *Rhizophagus*, a *Funneliformis* OTU (OTU806) was more abundant in seedlings grown after pea, compared to the other soil origins. For infestation with WCR, significant responses were observed for *Actinomucor* sp. (OTU135) and the family Mucoraceae in general, where *Actinomucor* sp. (OTU135) sequences were 2–6 times more abundant in infested maize rhizospheres compared to non-infested, depending on soil provenance (Supplementary Table 7). The fungal OTUs classified as *Trichoderma*, *Candida* and *Endogone*, as well as unclassified OTUs within the Agaricomycetes, Pezizomycetes and Ascomycota, were at least two times less abundant in maize rhizosphere grown in *Fusarium* infested soils (Supplementary Table 8). In contrast, the class Chytridiomycetes had 50% greater sequence abundance in samples from *Fusarium* infested soils.

### Functional diversity prediction

Predicted bacterial functional diversity, as a whole, did not respond to soil provenance, with no observable clustering of samples on the ordination space based on soil origin (Supplementary Figure 7). The community of predicted functional genes in samples originating from WCR infested samples, appear to differentiate from non-infested counterparts when maize plants were grown in soils preceded by either sunflower or maize (variation in axis 2). However, no consistent significant effects on abundance of predicted gene content or orthologous groups in response to infestation were observed across experiments.

**Figure 3:**
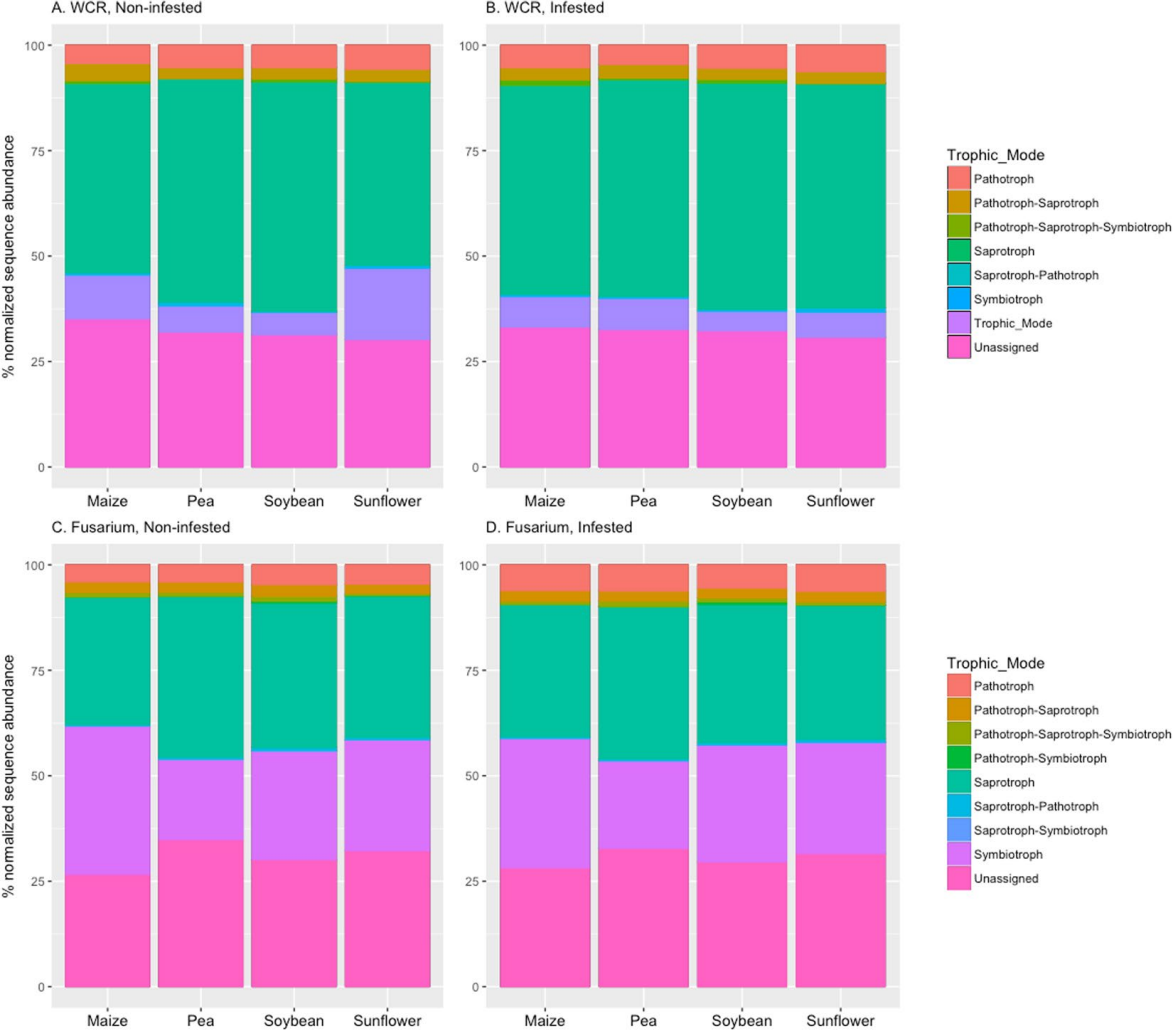
Relative abundance of fungal trophic modes recovered from the rhizosphere of maize grown in soils from four different four-year rotation sequences and exposed to infestation with western corn rootworm (**A,B**) and *Fusarium graminearum* (**C,D**). The taxa by sample matrix was compared against the FUNGuild database^42^. Normalized sequence abundance was aggregated by soil provenance (prior crop) and trophic mode, and presented as percent of the total normalized sequence abundance.

The predominant trophic mode of fungi recovered in this data set was saprotroph, ranging from 30 to 54% of normalized reads of non-infested samples, followed by symbiotroph (range 5–35%), in particular arbuscular mycorrhizal fungi, and pathotroph (range 4–6%; Fig. 3). However, the distribution of trophic modes across non-infested samples from different soil provenance differed. For instance, for samples preceded by pea and soybean, up to 10 times more saprotrophs than symbiotrophs were recovered from maize rhizosphere. Whereas two to four times more saprotrophs than symbiotrophs were recovered from samples preceded by sunflower and maize. Similarly, in non-infested samples preceded by maize or sunflower up to five times more symbiotroph sequences were recovered than pathotrophs. In the *Fusarium* experiment, a greater proportion of symbiotroph sequences were recovered, with as many as saprotroph sequences (1:1), in particular for samples preceded by maize. Depending on the experiment, and treatment combination, between 26–37% of the fungal OTUs were not assigned to a particular trophic mode. This is due to the lack of resolution in taxonomic assignment for these fungal OTUs and does not mean that these taxa are not functionally important.

### Relationship with plant vigor measurements

**Figure 4:**
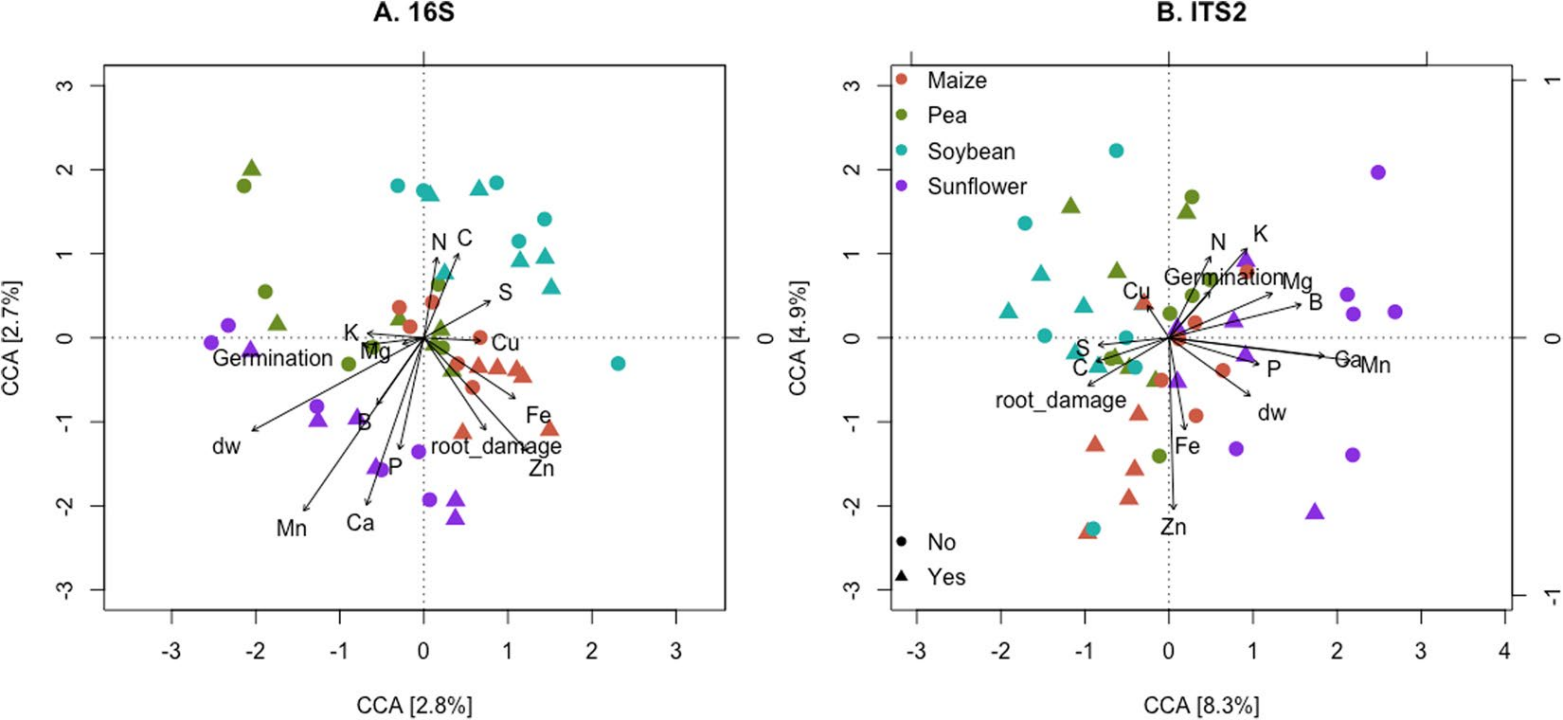
Relationship between maize-associated (**A**) bacterial (16S) and (**B**) fungal (ITS2) communities and seedling vigor measurements of maize plants grown in soils preceded by different crops under infestation with western corn rootworm. Ordination plots of analyzed samples were generated through canonical correspondence analysis (CCA) of microbial data against plant measurements, for n = 6 plant samples per soil provenance (color) and infestation (shape) treatment combination. The percentage of variation explained by each of the canonical axis is shown. Vectors display plant variables considered in the analysis. Plant nutrients: C, N, S, P, Cu, Fe, K, Zn, Ca, B, Mg, Mn. Germination refers to total germination at the time of sampling. Root_damage score was calculated based on WCR damage (see Material and Methods). dw, shoot dry weight.

Canonical correspondence analysis (CCA) was performed to evaluate correlations between plant vigor measurements and the structure of bacterial and fungal communities, in response to soil provenance and infestation. For CCA, the microbial community data was ordinated on axes resulting from the set of plant (i.e. explanatory) variables measured^48^. For the WCR experiment, plant variables explain 34% of the variation of both the bacterial (16S) and fungal (ITS2) community data; with the first two canonical axes explaining 5% and 13% of the total variation, for each microbial dataset (Fig. 4). The ordination plot of the bacterial communities recovered from the WCR experiment (Fig. 4A), reveals that for the first canonical axis shoot dry weight, germination, and certain plant elements (B, Mn, Ca and P) correspond in direction with communities from samples preceded by sunflower and pea; whereas plant S, Cu and C, are opposed in direction, relating with samples preceded by maize or soybean. Only samples preceded by maize exhibit some separation on the second canonical axis in relation to WCR infestation, and appear to be related with root damage and plant Zn and Fe. CCA of the WCR experiment fungal data shows separation between samples preceded by sunflower and soybean along the first canonical axis (Fig. 4B), which relate to shoot nutrient content (K, N, B, Mg, Mn, Ca, S, Cu, C) and shoot biomass. Samples originating from soils preceded by pea and maize cluster apart in the second canonical axis, with plant Zn and Fe content correlated with samples preceded by maize. As for the bacteria, clustering of samples in response to infestation is observed for samples preceded by maize, on the first canonical axis only.

**Figure 5:**
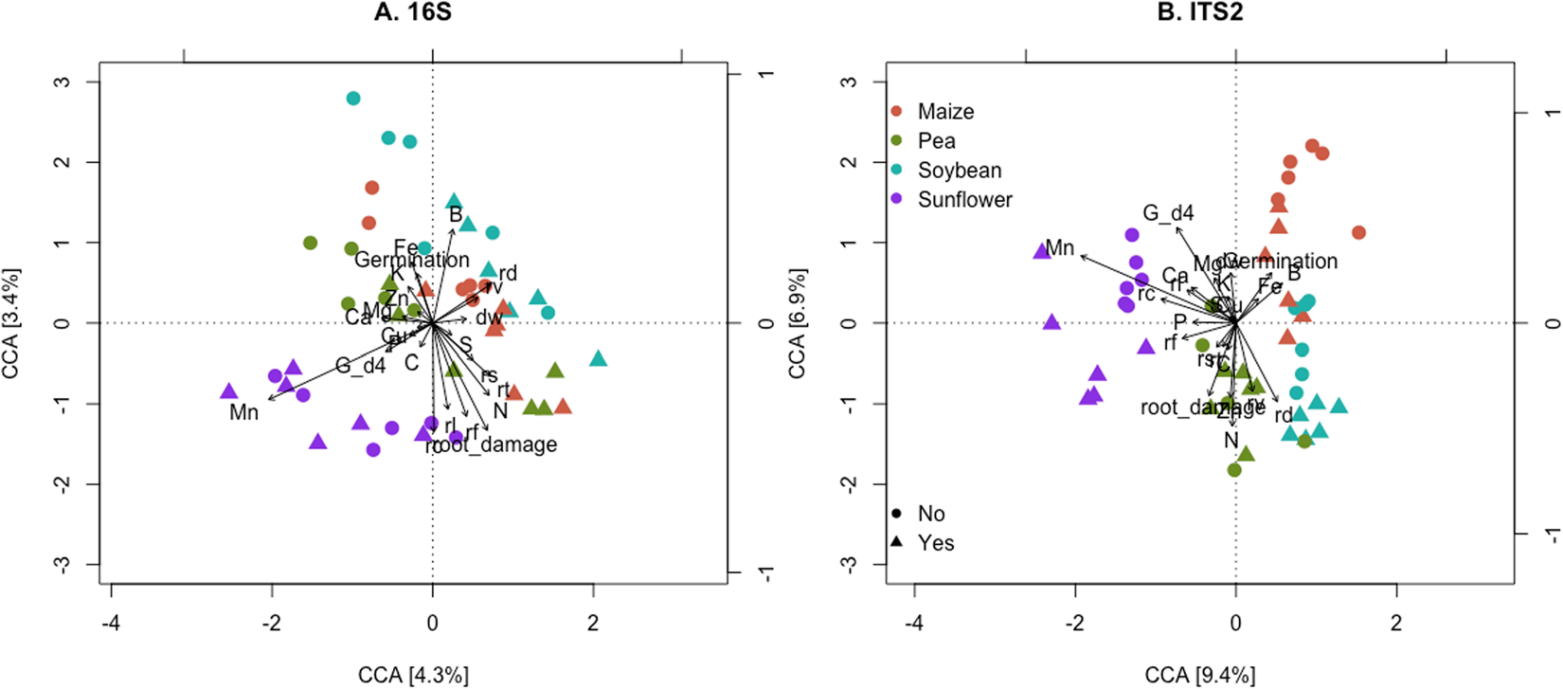
Relationship between maize-associated (**A**) bacterial (16S) and (**B**) fungal (ITS2) communities and seedling vigor measurements of maize plants grown in soils preceded by different crops under infestation with *F*. *graminearum*. Ordination plots of analyzed samples were generated through canonical correspondence analysis (CCA) of microbial data against plant measurements, for n = 6 plant samples per soil provenance (color) and infestation (shape) treatment combination. The percentage of variation explained by each of the canonical axis is shown. Vectors display plant variables considered in the analysis. Plant nutrients: C, N, S, P, Cu, Fe, K, Zn, Ca, B, Mg, Mn. Germination refers to total germination at the time of sampling, and G_d4, germination at day 4. Root_damage score was calculated based on % root damage (see Material and Methods). dw, shoot dry weight. Root architecture measures: rl = root length, rd = root diameter, rv = root volume, rs = root surface area, rf = number of root forks, rc = number of root crossings.

For the *Fusarium* experiment, plant variables explain 54% and 53% of the variation of the bacterial and fungal data; with the first two canonical axes explaining 7% and 16% of the total variation, respectively (Fig. 5). For the bacterial dataset (Fig. 5A), samples from soils preceded by sunflower cluster apart from the other three soil origins, and correlate with germination at day four, plant Mn, Cu and C; in contrast to, root diameter, root volume and plant B. The first canonical axis of the fungal CCA ordination (Fig. 5B) reveals three groups of samples, samples preceded by i) sunflower, ii) pea, and iii) maize and soybean. Plant variables correlating with samples preceded by sunflower include plant Mn, P, Ca, germination at day four, root length, root forks and root crossings. Samples from soils preceded by soybean and maize separate from each other along the second canonical axis, which correlate with plant C, N and Zn content as well as root damage (the latter with direction toward samples preceded by soybean and pea), and plant Fe, B and total germination (direction towards samples preceded by maize). Within samples preceded by maize and soybean, infested samples show some differentiation from non-infested along the second canonical axis.

## Discussion

The benefits of different four-year rotation sequences on subsequent maize seedling health and associated microbial communities were evaluated upon exposure to WCR or *F*. *graminearum*. Western corn rootworm infestation resulted in greater than 50% of total root damage, with no difference in root damage across soils from different rotation treatments (Table 1). In spite of the extent of root damage, only maize plants grown in soils preceded by pea had significantly lower (7%) shoot biomass in infested plants compared to non-infested. This finding is not consistent with our experimental hypothesis, as the rotation sequence ending in pea in the field tends to benefit subsequent maize yield. However, maize plants grown in infested soils preceded by pea and sunflower, had higher shoot biomass than plants grown after maize and soybean, regardless of infestation. Severe pruning by WCR has been previously associated with decreases in dry weight^49^, but root damage does not necessarily predict biomass and yield, since regrowth can occur in damaged roots^50^. In addition, multiple factors can affect maize responses to WCR damage. For instance, other studies have reported year-to-year variation in plant height or biomass responses to WCR infestation, as well as variations due to host genetics and differences in hybrid tolerance^51,52^. In addition, compensatory effects have been observed in maize biomass in response to WCR damage^53^, where biomass is greater in infested plants. In this study, compensatory effects were also observed, with infestation resulting in significantly higher C content in maize shoots, in particular for plants grown in soils preceded by maize or soybean (Supplementary Table 2). Consistent with other studies, WCR infestation resulted in reduced plant N, K, Ca, Mg^49^, as well as plant P, Mn and B. For P and K, pair-wise comparisons between infested and non-infested plants from the same preceding soil were non-significant when seedlings were grown after sunflower (and P only for maize). The impact of preceding crop on WCR larval stage or beetle emergence was not measured in this study, as our objective was to evaluate host growth and measure root damage before root re-growth occured. Presence of microorganisms can affect WCR larval development, as changes in the number of larvae in second or third instar, were observed in response to arbuscular mycorrhizal (AM) inoculation^54^. In that work, no differences in root biomass or damage were observed in AM-inoculated and WCR infested roots, compared to AM-inoculated and non-infested roots.

The effects of WCR infestation were detected for a small subset of fungal OTUs and bacterial genera. Previous studies have described effects of WCR infestation on bacterial and fungal community fingerprints^54,55^ as well as in the dominance of specific bacterial groups isolated from the rhizosphere of infested and non-infested maize^56^. For instance, Dematheis *et al*.^55^ identified a 16S gene marker (gel band), through denaturing gradient gel electrophoresis (DGGE) which increased in intensity in infested roots. The corresponding DGGE band was classified as *Acinetobacter calcoaceticus* (Pseudomonadales, Moraxellaceae). Consistent with Dematheis *et al*.^55^, in this work, sequences belonging to the genera *Acinetobacter* were 40 times more abundant in infested maize rhizospheres compared to non-infested (Supplementary Table 7). In addition, isolates of *Serratia* were previously preferentially isolated from WCR infested maize rhizospheres, compared to non-infested^56^. The Enterobacteriaceae OTU327, identified in this work, exhibited between 90–99% sequence identity to all *Serratia* isolates from Prischmann *et al*.^56^. OTU327 was also present at higher abundance in infested rhizospheres regardless of soil provenance, however differences were non-significant. In contrast, significant effects of infestation were observed for the Actinobacteria *Smaragdicoccus* and *Aeromicrobium*, as well as for taxa aggregated at the order level, the Enterobacteriales (family Enterobacteriaceae in particular) and Pseudomonadales (families Pseudomonadacea and Moraxellaceae) (Supplementary Table 7). Isolates from both Enterobacteriales and Pseudomonadales were also recovered from infested maize roots^56^. For fungi, significant responses to WCR infestation were observed for the genus *Actinomucor* (and the Mucoraceae family in general). *Actinomucor* sequences were 2–6 times more abundant in infested maize rhizosphere compared to non-infested, and the magnitude of the difference depends on soil provenance (Supplementary Table 7). Changes in fungal community fingerprints in response to WCR infestation have been reported^55^, however, no individual fungal taxa have been previously associated to WCR infested maize.

Soil infestation with *F*. *graminearum* resulted in significant differences in root damage and germination, and in the relative abundance of the *F*. *graminearum* OTU in maize rhizosphere. The average percent root damage, however, was under 3% and *F*. *graminearum* infestation did not affect dry weight. In addition, compensatory effects were observed on shoot N, P, K, S and Zn content, with shoots of *F*. *graminearum* infested plants having up to 12% more of a particular nutrient than non-infested plants. *F*. *graminearum* has been previously described as an important seed and seedling pathogen of maize^57^; however, under our experimental conditions, even though infestation and colonization was successful (up to three times higher sequence relative abundance in infested seedlings) root damage was low and no strong effects on germination were observed. The isolate used in this study, however, was obtained from symptomatic maize root tissue from South Dakota, and was characterized as the most aggressive in a greenhouse setting^27^. Factors contributing to *Fusarium* seedling blight development in maize include consistently low temperature and higher moisture, as well as host resistance^57,58^. It is likely that the warmer temperature ranges in our greenhouse, used to optimize maize development, in combination with hybrid genetics contributed to the low disease incidence in this experiment. The maize hybrid used in this study is described as providing “excellent agronomics”, very good tolerance to diseases and “good overall plant health”^59^.

Infestation with *F*. *graminearum* did affect relative abundance of individual fungal taxa. Species of *Trichoderma* (Hypocreaceae), a *Candida* species (Saccharomycetes), *Endogone* (Mucormycotina, Endogonales) and unknown taxa within the Pezizomycetes and Agaricomycetes had lower relative abundance in *F*. *graminearum* infested samples, regardless of soil origin. Taxa within these groups have been previously described as common endophytes in maize^60,61^, with groups such as *Trichoderma* and *Endogone* ^62^ potentially being beneficial to the plant host. Competition between species of *Trichoderma* and *Fusarium*, both in the rhizosphere and as saprophytes in plant residue, has also been observed in different cropping systems^63^. *Fusarium* infestation, however, had little consistent effects on the relative abundance of bacterial taxa recovered on maize seedling rhizosphere across soil provenance.

Soil provenance had greater impact in microbial community structure than biotic stress. Even after extensive root damage by WCR, the effect of soil provenance on bacterial and fungal community structure was significant and consistent across experiments (Figs 1 and 2). Lower fungal and bacterial diversity were found in the rhizosphere of maize seedlings following pea and soybean compared to maize and sunflower. The effect of preceding crop on fungal communities, however, was much larger compared to the effect on bacterial communities. In particular, the preceding crops sunflower and maize resulted in a significantly higher proportion of Glomeromycota sequences compared to pea and soybean. In this experiment, rotations ending with maize, pea and sunflower, share the other three crops in the four-year sequence hence potentially sharing some field history or legacy effect. The effects of field history on soil biota and microbial community structure have been detected up to 2 years after a change in management^16,17,64,65^. Similarly, rhizosphere-associated microbial communities of various plant species have shown to respond to differences in soil characteristics and management^22,66–68^.

Some variation is observed for the results of non-infested conditions between the WCR and *Fusarium* experiments. Experiments were designed in order to maximize maize growth (hence warmer greenhouse conditions), as well as to ensure detection of pest or pathogen damage. In addition, pest or pathogen inoculum was applied with a different methodology and substrate in each experiment. For the *Fusarium* experiment, maize seedlings were sampled two weeks after planting (vegetative stage 2), targeting early season damage. For WCR, four weeks were necessary to ensure that infested eggs reached to second or third instar and maximize root damage. Microbial and plant substrate, as well as inoculation method can affect resulting community structure and effects on plant health^69^. Similarly, plant development, is known to affect rhizosphere microbial community composition^70,71^, as microbial communities associated to plants are dynamic^72^.

The rotation sequence where sunflower preceded maize resulted in consistent responses from subsequent maize seedlings across experiments. In addition to greater shoot biomass, plant nutrient, and germination responses were also observed in maize seedlings grown after sunflower. These seedlings also had greater proportion of root length represented by the lowest diameter class (0–0.5 cm). As described by Lynch^73^, smaller root diameter results in lower tissue density, and greater efficiency for soil exploration. Each gram of root tissue can explore a greater soil volume. Furthermore, fine roots are cheaper to construct, penetrate finer soil pores and are short lived^73^, potentially benefiting maize seedling growth. Though not significant, under non-infested conditions, the number of root tips, crossings and forks, characteristics used to evaluate root architecture and branching patterns^74^, also had a tendency to be higher in plants grown in soils preceded by sunflower. Differences in root architecture, branching and diameter size class can also influence the extent of mycorrhizal colonization^75^. The mycorrhizal fungi represent the group of fungi which strongly responded to soil provenance in this experiment. Finally, sunflower as preceding crop had a distinct effect on microbial community composition in maize seedlings, compared to the other three preceding soils.

Overall, our results indicate that the preceding crop in a rotation affects the microbial community colonizing the maize rhizosphere, and influences maize seedling growth characteristics. A limited amount of evidence reflected crop-specific effects on the maize rhizosphere that influenced the response of the maize seedling to biological stressors. It is possible that the limited response of maize seedlings to the WCR infestation or *F*. *graminearum* infestation may reflect the relatively positive state of the soil microbiome in all soils which were collected from long term, no-till, diversified rotations, even after dilution. Benefits of crop rotation have long-been appreciated; understanding the specific effects of crop sequences and mechanisms that confer these effects in subsequent crops will promote increased adoption of favorable crop rotations.

## Electronic supplementary material

Supplementary Information

Supplementary tables 5-9
