# Supplementary material for: Previous crop and rotation history effects on maize seedling health and associated rhizosphere microbiome
Source: Sci Rep. 2017 Nov 16;7:15709. doi: 10.1038/s41598-017-15955-9 (PMC5691165; doi:10.1038/s41598-017-15955-9)
Supplement: Supplementary file 1 — Supplementary Information [file 41598_2017_15955_MOESM1_ESM.pdf]

Previous crop and rotation history effects on maize seedling health and associated rhizosphere microbiome

Maria-Soledad Benitez*, Shannon L. Osborne, R. Michael Lehman

USDA-ARS, North Central Agricultural Research Laboratory, Brookings, SD 57006

Supplementary Information

Supplementary Table 1. Primer sets and associated sequences used for MiSeq library preparation in this study.

**16S for MiSeq sequencing**			
**Primer name**	**Overhang (for Nextera kit indexing)**	**Gene-specific primer**	**References**
515f modified	TCGTCGGCAGCGTCAGATGTGTATAAGAGACAG	GTGYCAGCMGCCGCGGTAA	Walters et al 2015
806r modified	GTCTCGTGGGCTCGGAGATGTGTATAAGAGACAG	GGACTACNVGGGTWTCTAAT	
**ITS2 for MiSeq sequencing**			
**Primer name**	**Overhang (for Nextera kit indexing)**	**Gene-specific primer**	
ITS3mix1	TCGTCGGCAGCGTCAGATGTGTATAAGAGACAG	CATCGATGAAGAACGCAG	Tedersoo et al 2014
ITS3mix2	TCGTCGGCAGCGTCAGATGTGTATAAGAGACAG	CAACGATGAAGAACGCAG	
ITS3mix3	TCGTCGGCAGCGTCAGATGTGTATAAGAGACAG	CACCGATGAAGAACGCAG	
ITS3mix4	TCGTCGGCAGCGTCAGATGTGTATAAGAGACAG	CATCGATGAAGAACGTAG	
ITS3mix5	TCGTCGGCAGCGTCAGATGTGTATAAGAGACAG	CATCGATGAAGAACGTGG	
ITS3mix10	TCGTCGGCAGCGTCAGATGTGTATAAGAGACAG	CATCGATGAAGAACGCTG	
ITS4ngs	GTCTCGTGGGCTCGGAGATGTGTATAAGAGACAG	TCCTSCGCTTATTGATATGC	

References

Tedersoo, L. *et al*. Fungal biogeography. Global diversity and geography of soil fungi. *Science* **346**, 1256688 (2014).

Walters, W. *et al*. Improved Bacterial 16S rRNA Gene (V4 and V4-5) and Fungal Internal Transcribed Spacer Marker Gene Primers for Microbial Community Surveys. *mSystems* **1** (2015).

Supplementary Table 2. Nutrient content [a] (mg/g for macronutrients and µg/g for micronutrients) in dried shoot tissue of maize seedlings grown in soils preceded by four different four-year rotation sequences under infestation with western corn rootworm or *Fusarium graminearum*

Soil provenance	Infestation	Carbon			Nitrogen			Phosphorus			Potassium			Sulfur			Calcium			Magnesium			Zinc			Manganese			Boron			Copper			Iron	
**Western corn root worm**																																				
**Maize**	**No**	399.13	±5.33	B	12.54	±0.83	A	4.43	±0.31		49.60	±4.10	A	2.07	±0.12	A	3.82	±0.30	aA	2.80	±0.19	A	18.13	±1.92		66.06	±4.34	aA	26.04	±4.28	A	2.45	±1.53		60.58	±20.63
**Pea**	**No**	401.19	±9.83		12.29	±0.36	A	4.27	±0.48	A	48.03	±3.68	A	2.13	±0.22		3.57	±0.47	abA	2.65	±0.14	A	16.55	±2.63		63.45	±14.28	aA	23.23	±4.69	A	2.02	±0.87		70.34	±49.54
**Soybean**	**No**	403.27	±5.96	B	12.22	±0.82	A	4.17	±0.40	A	49.38	±2.28	A	2.00	±0.14		3.25	±0.24	b	2.78	±0.13	A	18.46	±4.4		47.61	±6.79	bA	21.24	±2.2	A	1.53	±1.01		74.89	±75.58
**Sunflower**	**No**	402.32	±6.01		12.03	±0.4	A	4.30	±0.31		49.02	±3.04		1.93	±0.15		3.77	±0.25	aA	2.73	±0.22	A	19.84	±6.32		66.04	±6.02	aA	23.87	±3.68	A	2.13	±1.07	B	74.66	±75.34
**Maize**	**Yes**	405.47	±6.44	A	10.75	±0.32	B	4.22	±0.33	a	44.33	±3.77	B	1.95	±0.14	B	3.22	±0.35	abB	2.37	±0.19	B	20.63	±4.80		49.80	±4.28	bB	16.69	±1.13	abB	2.70	±1.51		50.69	±21.72
**Pea**	**Yes**	410.13	±3.36		10.74	±0.44	B	3.77	±0.21	bB	44.08	±1.80	B	1.95	±0.15		3.03	±0.23	bcB	2.27	±0.14	B	15.19	±3.25		42.00	±2.72	cB	15.98	±1.82	bcB	3.04	±2.98		40.39	±15.86
**Soybean**	**Yes**	411.50	±7.48	A	11.31	±0.62	B	3.57	±0.65	bB	43.53	±3.62	B	1.92	±0.20		2.80	±0.49	c	2.22	±0.23	B	16.50	±2.35		36.89	±7.05	cB	14.87	±2.27	cB	4.31	±8.16		58.86	±20.99
**Sunflower**	**Yes**	402.98	±5.63		10.80	±0.52	B	4.15	±0.25	a	46.07	±3.14		1.83	±0.29		3.38	±0.24	aB	2.42	±0.13	B	17.73	±3.52		56.94	±3.34	aB	18.93	±3.26	aB	2.80	±0.66	A	51.60	±22.74
	Soil Effect							*									**									***			**			*				
	Inoculation Effect	**			***			**			***			**			***			***						***			***							
F. graminearum																																				
**Maize**	**No**	409.29	±2.39	bc	13.11	±0.67	B	4.85	±0.3	a	51.65	±1.76	ab	3.02	±0.15		4.57	±0.29		2.85	±0.14		16.91	±0.82	bB	102.14	±14.33	b	47.95	±12.06		3.40	±0.88		58.66	±8.34
**Pea**	**No**	412.16	±1.72	aA	13.71	±0.72	B	4.43	±0.31	bB	47.28	±4.37	cB	2.88	±0.44	B	4.43	±0.58		2.65	±0.34		20.55	±3.37	a	94.19	±12.32	b	38.87	±3.49		2.93	±1.12		56.23	±14.72
**Soybean**	**No**	407.36	±2.15	cB	13.35	±0.89	B	4.88	±0.58	a	52.38	±5.32	a	2.93	±0.43		4.48	±0.58		2.70	±0.31		18.14	±0.67	aB	97.15	±12.39	b	45.78	±8.37		2.64	±1.64		54.42	±13.55
**Sunflower**	**No**	410.42	±1.63	ab	13.42	±0.67	B	4.92	±0.29	aB	50.67	±1.87	bc	2.93	±0.33		4.65	±0.42		2.83	±0.24		17.91	±1.29	abB	130.63	±10.25	a	41.04	±5.84		2.72	±0.76		56.01	±10.34
**Maize**	**Yes**	408.60	±4.07		15.13	±0.55	A	4.58	±1.24		47.03	±11.46		3.02	±0.88		4.25	±1.06		2.57	±0.63		18.38	±4.8	A	91.74	±28.4	b	43.08	±13.57	b	2.49	±0.87		52.79	±21.69
**Pea**	**Yes**	408.89	±1.01	B	15.08	±0.75	A	5.38	±0.31	A	54.57	±2.46	A	3.50	±0.13	A	4.73	±0.26		2.83	±0.1		21.26	±5.26		95.57	±7.6	b	39.27	±4.89	b	3.56	±1.00		62.07	±28.04
**Soybean**	**Yes**	409.23	±1.17	A	15.16	±0.29	A	5.08	±0.66		51.27	±6.48		3.18	±0.50		4.42	±0.51		2.70	±0.36		20.65	±1.00	A	88.87	±9.65	b	50.61	±6.21	a	2.81	±0.56		73.63	±41.85
**Sunflower**	**Yes**	407.63	±4.17		14.92	±0.52	A	5.37	±0.66	A	51.20	±4.5		3.17	±0.47		4.70	±0.51		2.75	±0.26		19.54	±1.25	A	138.47	±27.45	a	42.72	±6.41	b	3.04	±0.90		53.16	±4.35
	Soil Effect																						*			***			**							
	Inoculation Effect				***			***			*			**									***													

[a] Value represents mean of n=8 pots per soil origin and infestation treatment combination, ± standard deviation. Means followed by different letter are significantly different at *p*< 0.1 after Kruskal-Wallis test. Comparisons between rotation sequences, within infestation level are shown by lower case letters. Comparisons between infested and non-infested counterparts of the same rotation sequence are shown in upper case letters. Absence of letters mean no significance was detected. Significant responses to soil provenance (soil effect) and infestation are shown at <0.1 (*), <0.05(**) and <0.001(***).

Supplementary Table 3a. Illumina MiSeq sequencing summary results for 16S and ITS2 amplicon libraries

Marker	Sequences per marker	Paired-reads	Sequences used for OTU calling[a]	OTUs[b]	Treatments only sequences[c]	Treatments only OTUs[c]
16S	19,148,934	6,598,118	6,023,754	9,015	4,224,557	7,141
ITS2	13,282,922	2,821,329	1,541,454	1,505	839,315	1,023

[a] PF reads (passed filtered reads)

[b] paired-end sequences >200 nucleotides, quality trimmed, following usearch 8 pipeline and chimera checking

[c] bacterial or fungal (and oomycete) origin only

Supplementary Table 3b. Summary of number of OTUs and sequences obtained for each gene region and experiment before and after low abundance filtering

	All OTUs		Filtering of low abundance OTUs				
	Samples	OTUs	OTUs	Sequences	Range	Median	Mean
**16S**							
WCR	64	6,323	3,365	954,110	8,993-22,643	14,768	14908
Fusarium	64	6,676	4,079	1,547,068	6,256-76,631	2,2694	24173
**ITS2**							
WCR	64	622	218	66,586	147-4,323	696	1040
Fusarium	62	888	508	435,661	744-19,118	7063	7027

Supplementary Table 4. Relative abundance[a] of *Fusarium graminearum* OTU (Fg_OTU4) recovered through Illumina MiSeq sequencing of ITS2 amplicon libraries, from maize seedling rhizospheres growing in soils preceded by four different four-year rotation sequences with and without infestation

	Non-infested								Infested								Effects	
	**Maize**		**Pea**		**Soybean**		**Sunflower**		**Maize**		**Pea**		**Soybean**		**Sunflower**		**Soil origin**	**Infestation**
**Fg_OTU4**	2.58	B	2.45	B	4.33	B	3.86	B	11.82	A	12.18	A	12.15	A	12.04	A		***

[a] Values represents mean normalized abundance of ITS2 OTU4 recovered from n=8 maize roots (and associated soil). Samples were individually processed for ITS2 amplicon sequencing. Means followed by different letter are significantly different at $p < 0.1$ after Kruskal-Wallis test. Comparisons between rotation sequences, within infestation level are shown by lower case letters. Comparisons between infested and non-infested counterparts of the same rotation treatment are shown in upper case letters. Absence of letters mean no significance was detected. Significant effects of soil provenance or infestation are shown as * p<0.1, ** p<0.05, *** p<0.001

Supplementary Tables 5 and 6. Relative abundance of bacterial amplicons recovered from maize rhizosphere of seedlings growing in soils preceded by four different four-year rotation sequences and exposed to western corn root worm or *Fusarium graminearum* infestation. Suppl. Table 5 summarizes all bacterial phyla recovered and Suppl Table 6 bacteria at different taxonomic rank showing consistent responses to soil provenance across experiments. *See Excel file.*

Supplementary Table 7. Relative sequence abundance of bacterial and fungal taxa recovered from maize rhizosphere of seedlings growing in soils preceded by four different four-year rotation sequences which respond to western corn root worm infestation. Differential abundance was tested with data aggregated at different levels of the taxonomic hierarchy. Taxa are arranged from highest to lowest taxonomic rank when differences were observed at different levels. *See Excel file.*

Supplementary Table 8. Relative sequence abundance of bacterial and fungal taxa recovered from maize rhizosphere of seedlings growing in soils preceded by four different four-year rotation sequences which respond to infestation with *Fusarium graminearum*. Differential abundance was tested with data aggregated at different levels of the taxonomic hierarchy. Taxa are arranged from highest to lowest taxonomic rank when differences were observed at different levels. *See Excel file.*

Supplementary Table 9. Relative sequence abundance of fungal taxa recovered from maize rhizosphere of seedlings growing in soils preceded by four different four-year rotation sequences and exposed to western corn root worm or *Fusarium graminearum* infestation. Differential abundance was tested with data aggregated at different levels of the taxonomic hierarchy. Suppl. Table 9 summarizes all fungal phyla recovered as well as fungal taxa at different taxonomic rank showing consistent responses to soil provenance across experiments. *See Excel file.*

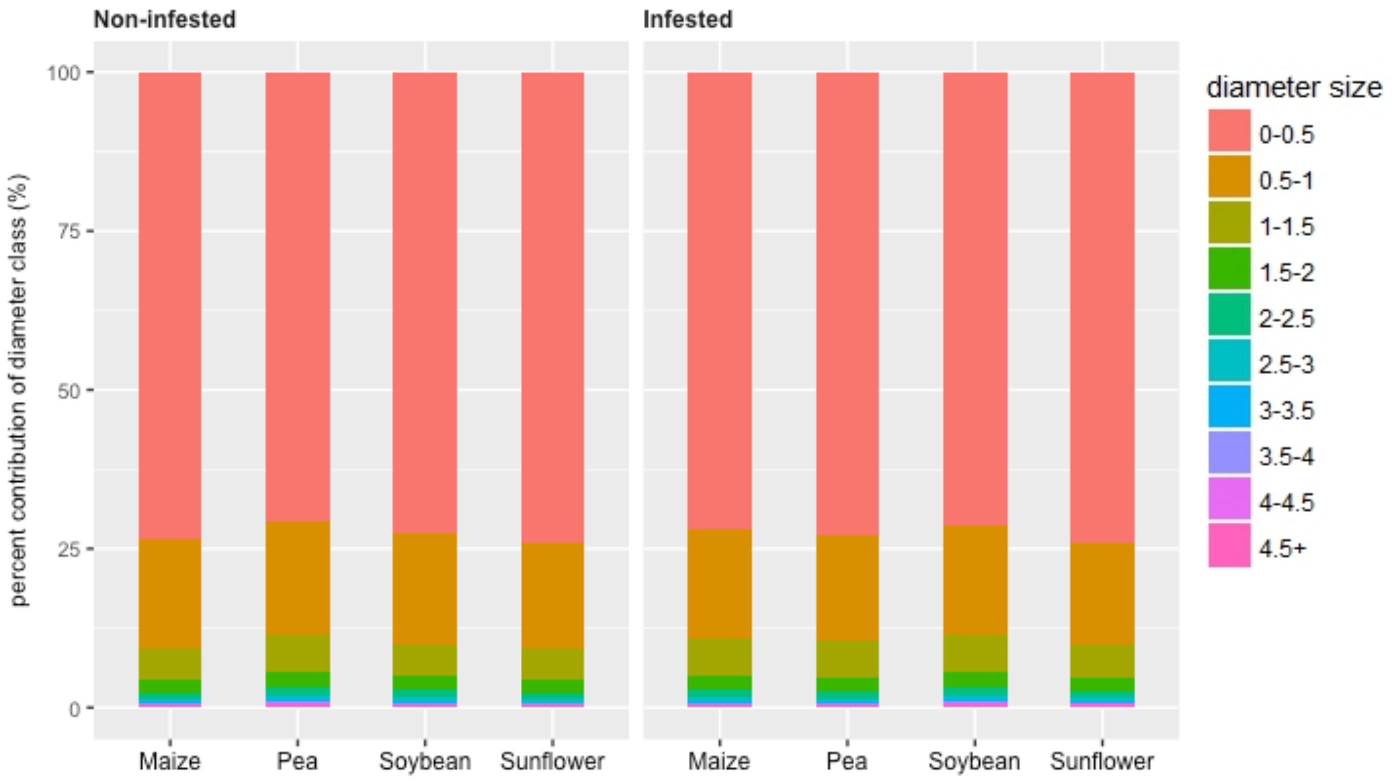

Supplementary Figure 1. Percent contribution of root length for different root diameter size classes (mm). Maize seedlings were grown in soils from four different four-year rotation sequences under infestation with *Fusarium graminearum* and roots were scanned and analyzed using WinRhizo software. Cumulative percent for each diameter class size is shown per soil provenance and infestation combination.

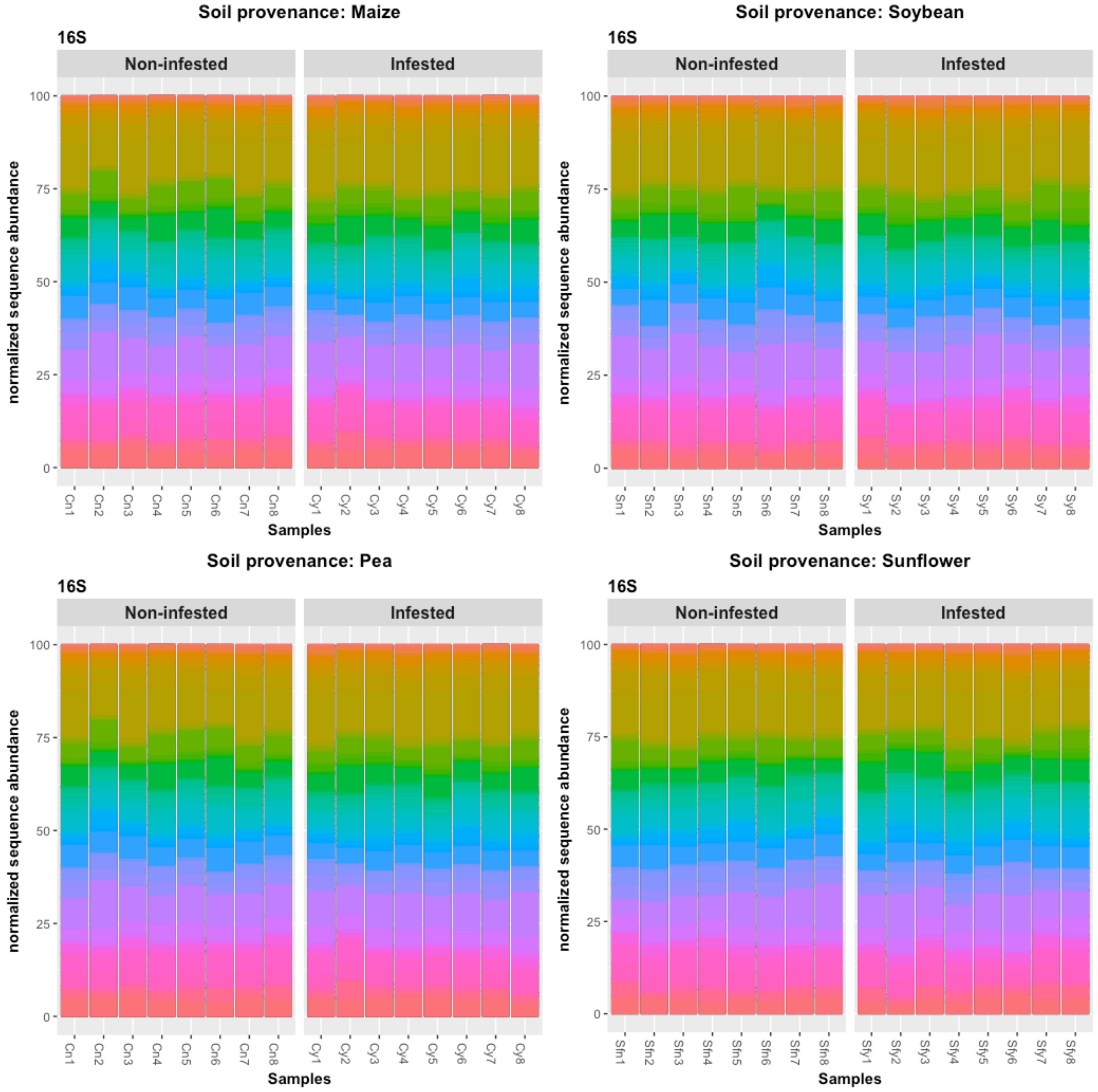

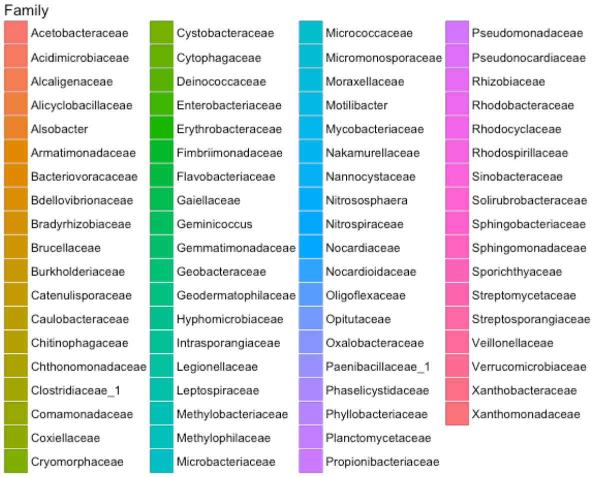

Supplementary Figure 2. Relative abundance of bacterial (16S) sequences from individual studied samples, aggregated at the family level. Sequences were recovered from the rhizosphere of maize seedlings grown in soils from four different four-year rotation sequences under infestation with western corn rootworm.

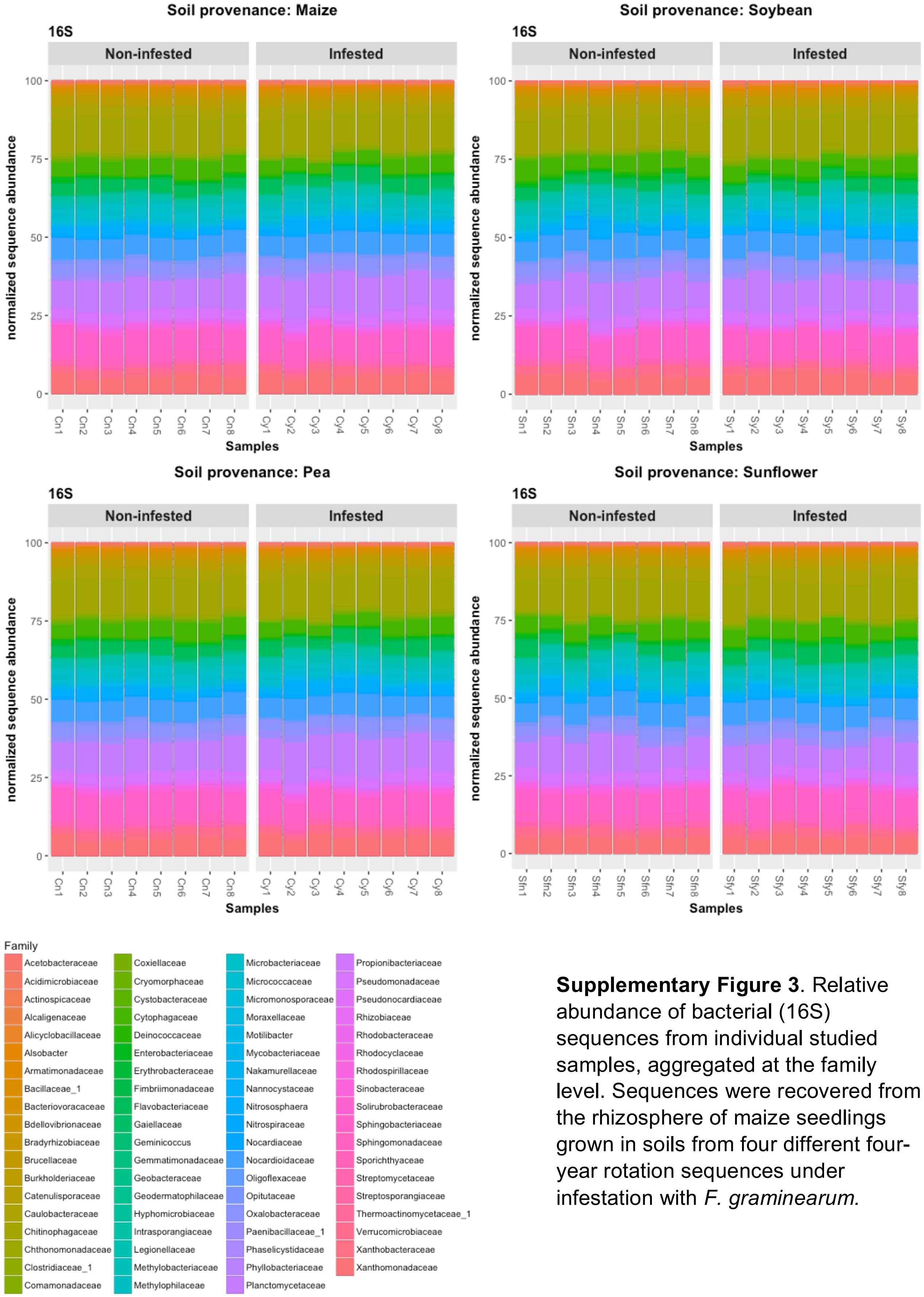

Supplementary Figure 3. Relative abundance of bacterial (16S) sequences from individual studied samples, aggregated at the family level. Sequences were recovered from the rhizosphere of maize seedlings grown in soils from four different four-year rotation sequences under infestation with *F. graminearum.*

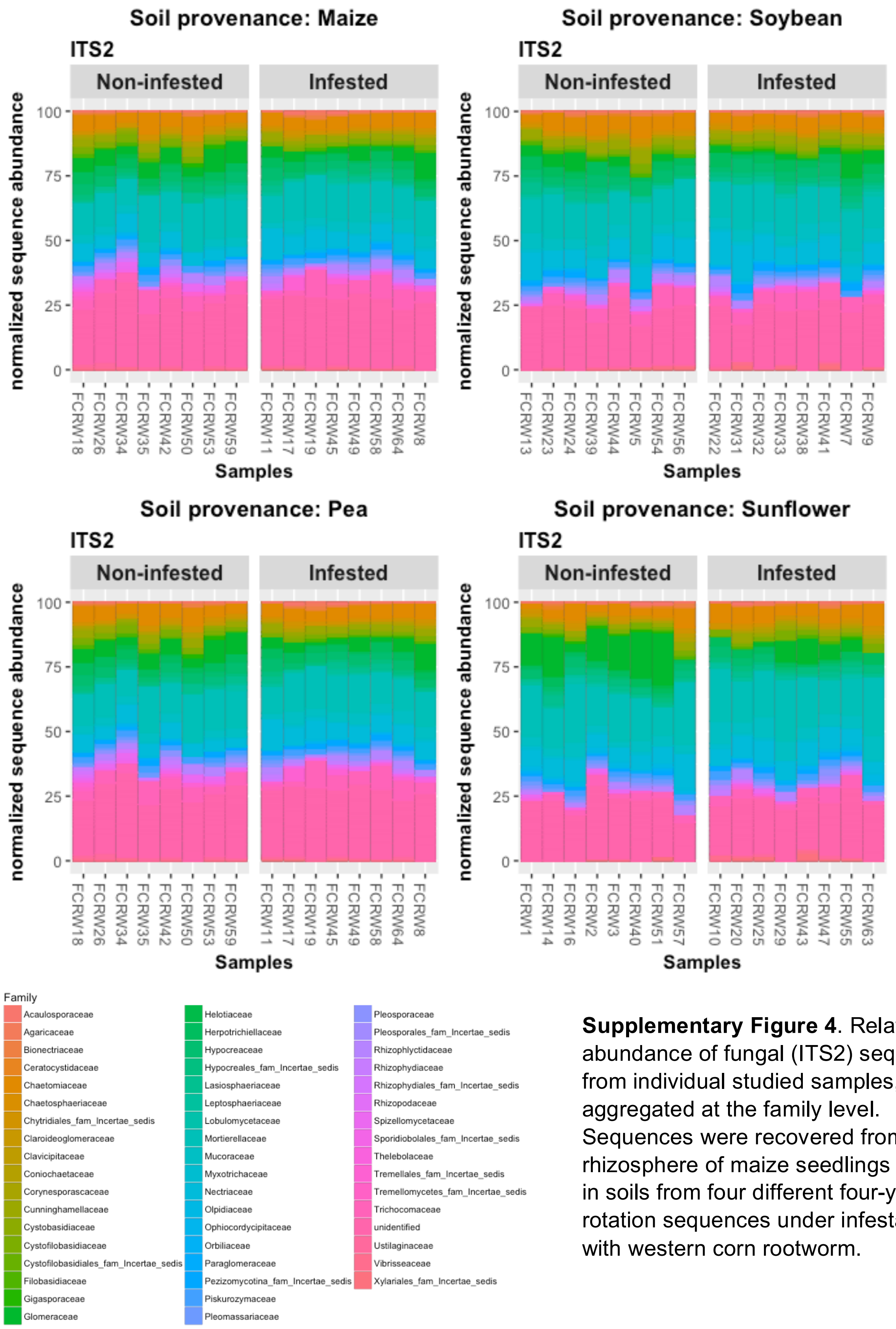

Supplementary Figure 4. Relative abundance of fungal (ITS2) sequences from individual studied samples, aggregated at the family level. Sequences were recovered from the rhizosphere of maize seedlings grown in soils from four different four-year rotation sequences under infestation with western corn rootworm.

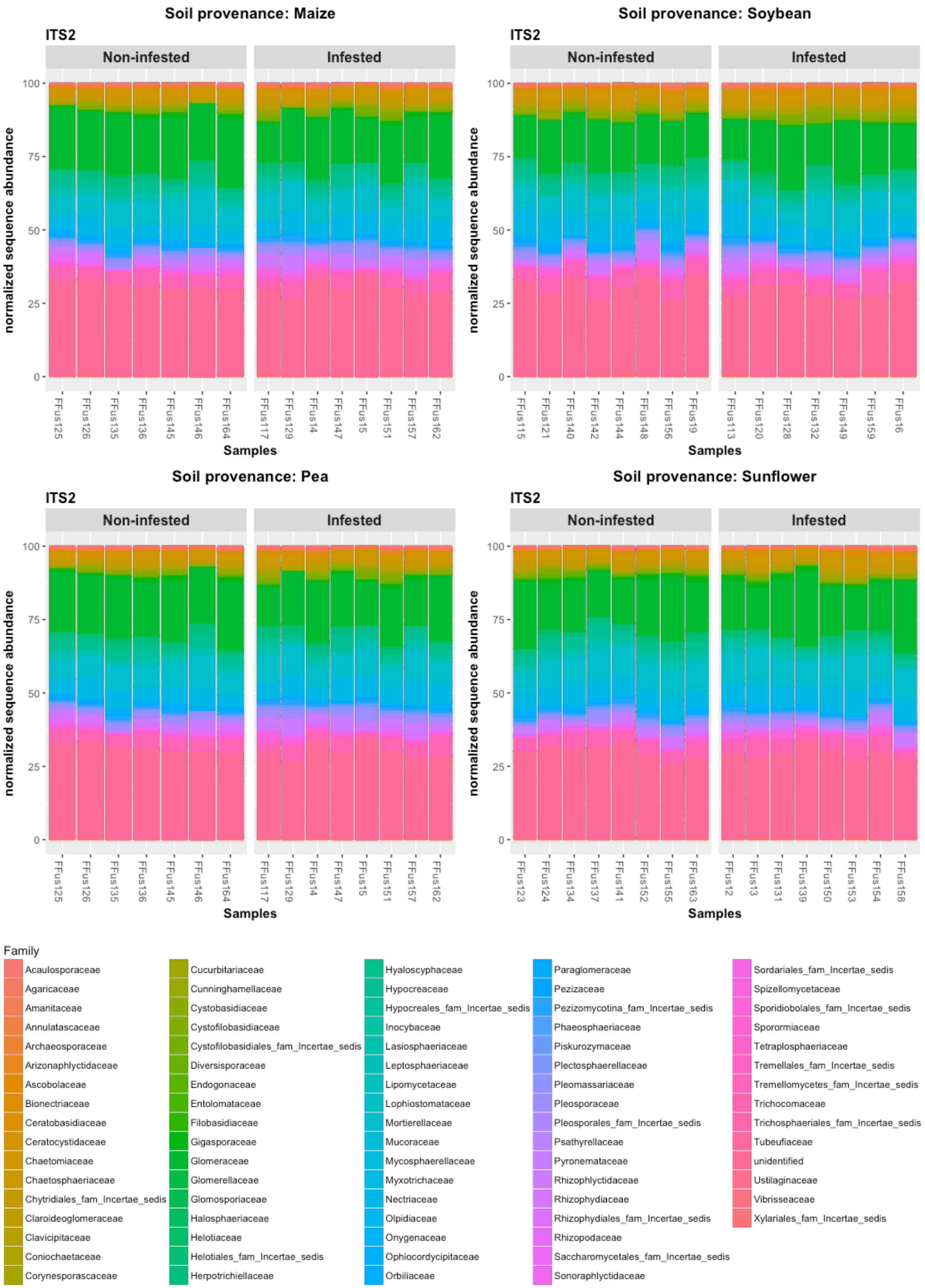

Soil provenance: Maize
ITS2
Non-infested
Infested
normalized sequence abundance
0
25
50
75
100
FFus125
FFus126
FFus135
FFus136
FFus145
FFus146
FFus164
FFus117
FFus129
FFus14
FFus147
FFus15
FFus151
FFus157
FFus162
Samples
Soil provenance: Soybean
ITS2
Non-infested
Infested
normalized sequence abundance
0
25
50
75
100
FFus115
FFus121
FFus140
FFus142
FFus144
FFus148
FFus156
FFus19
FFus113
FFus120
FFus128
FFus132
FFus149
FFus159
FFus16
Samples
Soil provenance: Pea
ITS2
Non-infested
Infested
normalized sequence abundance
0
25
50
75
100
FFus125
FFus126
FFus135
FFus136
FFus145
FFus146
FFus164
FFus117
FFus129
FFus14
FFus147
FFus15
FFus151
FFus157
FFus162
Samples
Soil provenance: Sunflower
ITS2
Non-infested
Infested
normalized sequence abundance
0
25
50
75
100
FFus123
FFus124
FFus134
FFus137
FFus141
FFus152
FFus155
FFus163
FFus12
FFus13
FFus131
FFus139
FFus150
FFus153
FFus154
FFus158
Samples
Family
Acaulosporaceae
Agaricaceae
Amanitaceae
Annulatascaceae
Archaeosporaceae
Arizonaphlyctidaceae
Ascobolaceae
Bionectriaceae
Ceratobasidiaceae
Ceratocystidaceae
Chaetomiaceae
Chaetosphaeriaceae
Chytridiales_fam_Incertae_sedis
Claroideoglomeraceae
Clavicipitaceae
Coniochaetaceae
Corynesporascaceae
Cucurbitariaceae
Cunninghamellaceae
Cystobasidiaceae
Cystofilobasidiaceae
Cystofilobasidiales_fam_Incertae_sedis
Diversisporaceae
Endogonaceae
Entolomataceae
Filobasidiaceae
Gigasporaceae
Glomeraceae
Glomerellaceae
Glomosporiaceae
Halosphaeriaceae
Helotiaceae
Helotiales_fam_Incertae_sedis
Herpotrichiellaceae
Hyaloscyphaceae
Hypocreaceae
Hypocreales_fam_Incertae_sedis
Inocybaceae
Lasiosphaeriaceae
Leptosphaeriaceae
Lipomycetaceae
Lophiostomataceae
Mortierellaceae
Mucoraceae
Mycosphaerellaceae
Myxotrichaceae
Nectriaceae
Olpidiaceae
Onygenaceae
Ophiocordycipitaceae
Orbiliaceae
Paraglomeraceae
Pezizaceae
Pezizomycotina_fam_Incertae_sedis
Phaeosphaeriaceae
Piskurozymaceae
Plectosphaerellaceae
Pleomassariaceae
Pleosporaceae
Pleosporales_fam_Incertae_sedis
Psathyrellaceae
Pyronemataceae
Rhizophlyctidaceae
Rhizophydiaceae
Rhizophydiales_fam_Incertae_sedis
Rhizopodaceae
Saccharomycetales_fam_Incertae_sedis
Sonoraphlyctidaceae
Sordariales_fam_Incertae_sedis
Spizellomycetaceae
Sporidiobolales_fam_Incertae_sedis
Sporormiaceae
Tetraplosphaeriaceae
Tremellales_fam_Incertae_sedis
Tremellomycetes_fam_Incertae_sedis
Trichocomaceae
Trichosphaeriales_fam_Incertae_sedis
Tubeufiaceae
unidentified
Ustilaginaceae
Vibrisseaceae
Xylariales_fam_Incertae_sedis

Supplementary Figure 5. Relative abundance of fungal (ITS2) sequences from individual studied samples, aggregated at the family level. Sequences were recovered from the rhizosphere of maize seedlings grown in soils from four different four-year rotation sequences under infestation with *F. graminearum*.

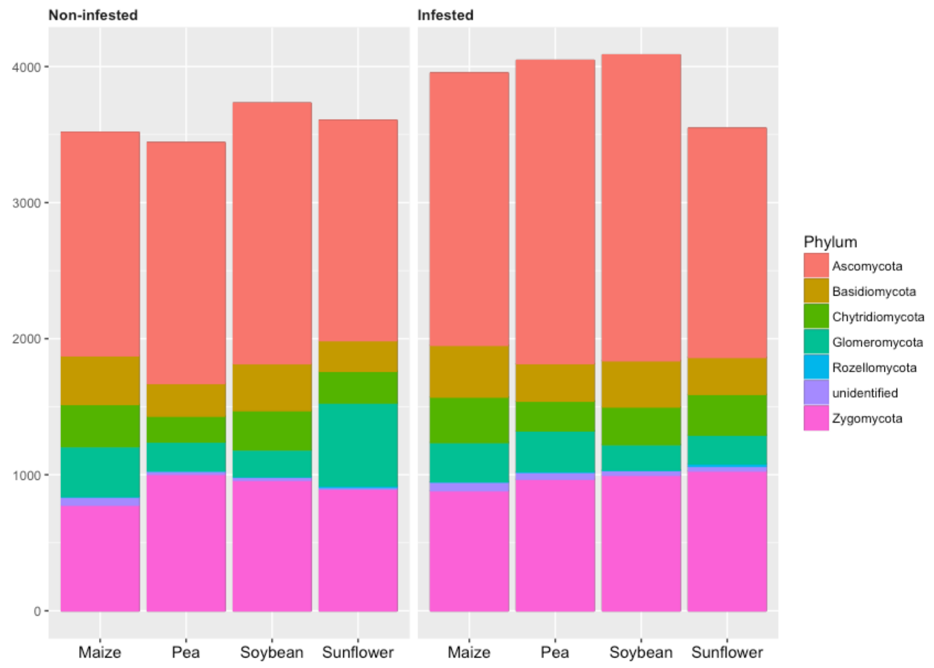

A.

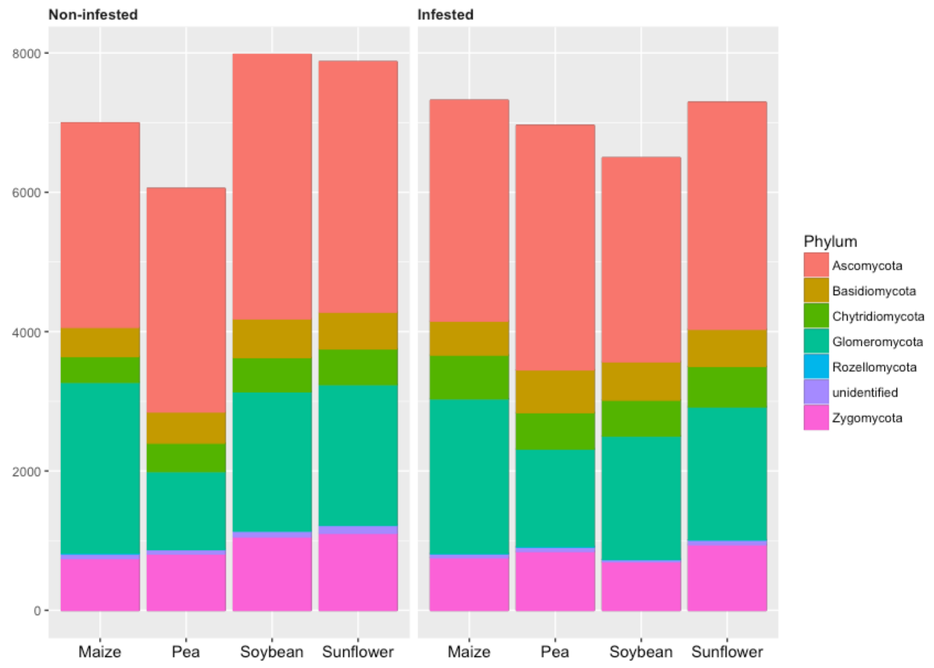

B.

Supplementary Figure 6. Relative abundance of fungal (ITS2) sequences aggregated at the phylum level. Sequences were recovered from the rhizosphere of maize seedlings grown in soils from four different four-year rotation sequences under infestation with western corn root worm (A) and infestation with *Fusarium graminearum* (B).

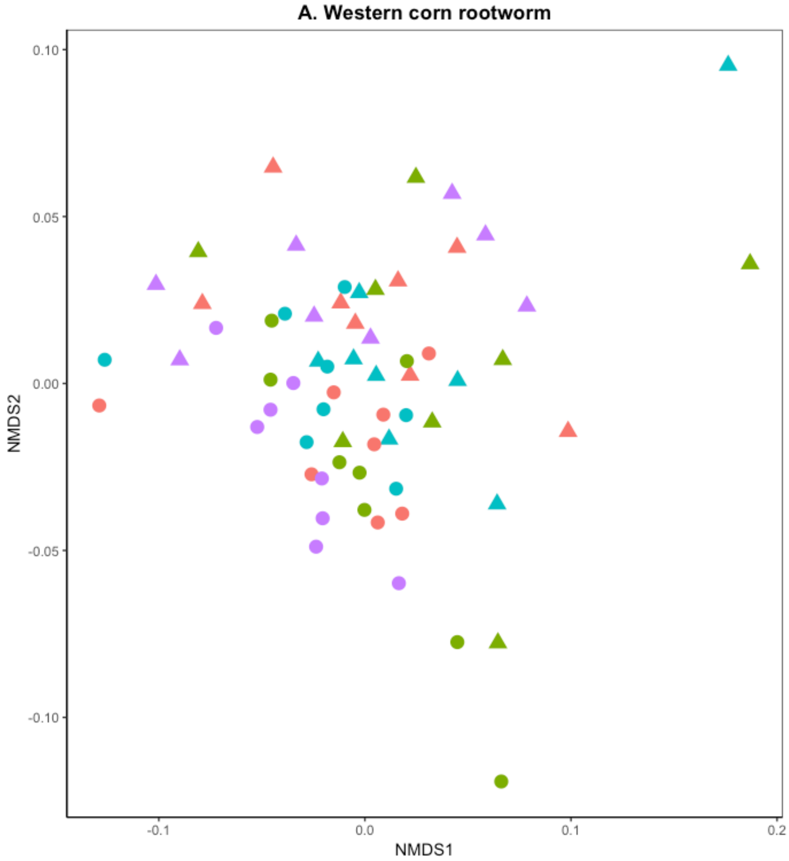
A. Western corn rootworm
NMDS2
NMDS1
0.10
0.05
0.00
-0.05
-0.10
-0.1
0.0
0.1
0.2

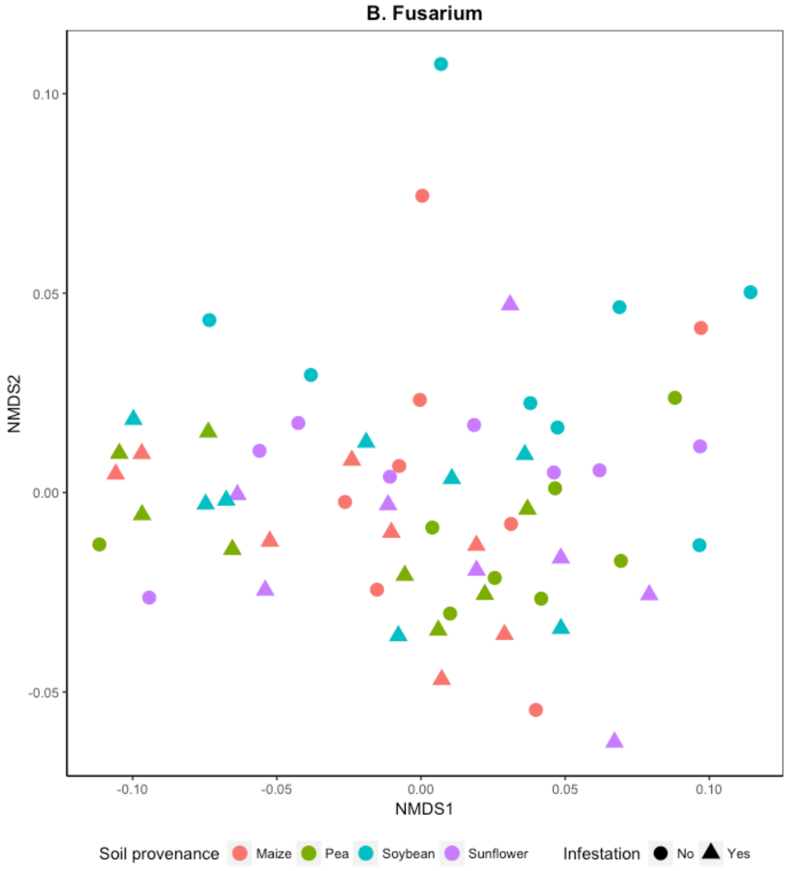
B. Fusarium
NMDS2
NMDS1
0.10
0.05
0.00
-0.05
-0.10
-0.05
0.00
0.05
0.10
Soil provenance
Maize
Pea
Soybean
Sunflower
Infestation
No
Yes

Supplementary Figure 7. Soil provenance and infestation effects on predicted metagenome gene content of rhizosphere-associated bacteria recovered from maize seedlings grown in soils from different four-year rotation sequences. Metagenome content prediction was performed using PICRUSt (Langille et al., 2013) after closed reference OTU clustering of 16S amplicon sequence. Ordination was performed based on non-metric multidimensional scaling (NMDS) of Bray-Curtis distance matrices for all samples.

Supplementary Methods.

Pipeline used for sequence processing, OTU calling and preparation of OTU by sample tables

#unless noted, the same pipeline was used for 16S and ITS2 sequences, replacing the first letter of the file name from b to f

#place all fastq files in one folder
cp B*/Data/Intensities/BaseCalls/*.fastq.gz ~/Documents/GH16bact_fastqfiles

#merge paired reads (after decompression of .gz files)
usearch -fastq_mergepairs *_R1_*.fastq -relabel @ -fastqout bmerged.fq

#trimming gene specific primers after merging and removal of sequences without primers
#for 16S sequences
~/.local/bin/cutadapt -g GTGYCAGCMGCCGCGGTAA -a ATTAGAWACCCBNGTAGTCC -e 0.1 --untrimmed-output=no_trim_bmerged.fastq --match-read-wildcards -o bmerged_trimm.fastq bmerged.fq

#for ITS2 sequences
~/.local/bin/cutadapt -g CAHCGATGAAGAACGYDG -a GCATATCAATAAGCGSAGGA -e 0.1 --untrimmed-output=no_trim_fbmergeded.fastq --match-read-wildcards -o fbmerged_trimm.fastq no_trim_bmerged.fastq

#OTU calling pipeline
usearch -fastq_filter bmerged_trimm.fastq -fastq_maxee 0.5 -fastq_minlen 200 -relabel Filt -fastaout bcfiltered.fa

usearch -derep_fulllength bfiltered.fa -relabel Uniq -sizeout -fastaout bcuniques.fa

usearch -cluster_otus buniques.fa -minsize 2 -otus bcotus.fa -relabel Otu -sizeout -sizein

usearch -uchime_ref bcotus.fa -db refdb.fa -strand plus -minh 1.0 -nonchimeras bcotus_nonch.fa \
  -uchimeout otus.uchime -uchimealns otus.aln

usearch -usearch_global bmerged_trimm.fastq -db bcotus_nonch.fa -strand plus -id 0.97 -otutabout botutab.txt -biomout botutab.json

usearch -makeudb_utax refdb.fa -output brefdb.udb -taxconfsin 250.tc

usearch -utax bcotus_nonch.fa -db brefdb.udb -utaxout butax.txt -strand both -rdpout butaxrdp.txt -alnout baln.txt

#for ITS2 taxonomy assignment use Qiime's assign taxonomy and full Qiime-formatted Unite database
assign_taxonomy.py -i fcotus_treatments_seq.fasta –r unite_ref/sh_refs_qiime_ver7_97_s_20.11.2016.fasta -t unite_ref/sh_taxonomy_qiime_ver7_97_s_20.11.2016.txt -m blas

#manually revise file and identify non-target OTUs for filtering. Incorporate into Qiime for filtering of non-target OTUs, low abundance OTUs, splitting into individual experiments and sequence count normalization

filter_otus_from_otu_table.py -i fcotush.biom -o fungi_only_fcotu_table -e otus_to_remove.txt

```
filter_samples_from_otu_table.py -i fungi_only_fcotu_table -o fungi_treat_only_table --sample_id_fp
control_samples.txt --negate_sample_id_fp

biom convert -i fungi_treat_only_table -o Fungi_otus_treatments_table_new.txt --to-tsv  #treatment only, sequence
count file for alpha diversity analysis (R, phyloseq)

split_otu_table.py -i fungi_treat_only_table -m mapping_ITS_treat.csv -f Experiment -o by_experiment

#this point forward, for each gene region files were split per experiment (WCR, Fusarium 1 and Fusarium 2)
filter_otus_from_otu_table.py -i fungi_treat_only_table__Experiment_FUS1_0116.biom -n 10 -s 5 -o
fcotus_filt_Jan2017_Fus1

filter_samples_from_otu_table.py -i fcotus_filt_Jan2017_Fus1 -n 5 -o fcotus_filt_Jan2017_Fus1ed

normalize_table.py -i fcotus_filt_Jan2017_Fus1ed -o Fus1_F_filt_norm -a CSS -s --output_CSS_statistics

biom convert -i Fus1_F_filt_norm -o F1_F_filt_CSSnew.txt --to-tsv #normalized file used for beta diversity and other
statistical analyses in R (phyloseq and vegan)

#for Picrust analysis: require greengenes identification, obtained through Qiime's closed reference OTU picking
#perform pick closed reference otus in qiime using the defaults (as in picrust.github.io otu picking tutorial)
echo "pick_otus:enable_rev_strand_match True" >> otu_picking_params_97.txt

echo "pick_otus:similarity 0.97" >> otu_picking_params_97.txt

pick_closed_reference_otus.py -i bcfiltered.fasta -p otu_picking_picking_params_97.txt -o bactc_cr_otus

# manually revise file and identify non-target OTUs for filtering, filter low abundance OTUs as above
filter_samples_from_otu_table.py -i otu_table.biom --sample_id_fp bcsample_list.txt -o otu_table_filtered

filter_otus_from_otu_table.py -i otu_table_filtered -o otu_table_bact_only_filt -n 10 -s 5 -e closedref_nobact_list.txt

#convert to biom json format for compatibility with picrust and to text file for comparison with results of usearch OTU
picking
biom convert -i otu_table_bact_only_filt -o bact_only_table_f_json.biom --table-type "OTU table" --to-json

biom convert -i otu_table_bact_only_filt -o bact_only_table_f.txt --to-tsv --header-key taxonomy

#run Picrust, integrated into bioBakery
normalize_by_copy_number.py -i bact_only_table_json.biom -o normalized_apr06.biom

predict_metagenomes.py -i normalized_apr06.biom -o apr_metagenome_predictions.biom -a apr_nsti_per_sample.tab
--with_confidence  #-f option generates a text version of the file to be integrated into R (phyloseq, vegan)

categorize_by_function.py -i apr_metagenome_predictions.biom -o apr_by_function_Kegg.txt -c KEGG_Pathways -l 3
```

```
categorize_by_function.py -i apr_metagenome_predictions.biom -o apr_by_Kegg_1.txt -c KEGG_Pathways -l 1
```
